# Histone H3K9 Lactylation Confers Temozolomide Resistance in Glioblastoma via LUC7L2‐Mediated MLH1 Intron Retention

**DOI:** 10.1002/advs.202309290

**Published:** 2024-03-13

**Authors:** Qu Yue, Zhao Wang, Yixiong Shen, Yufei Lan, Xiangyang Zhong, Xin Luo, Tao Yang, Manqing Zhang, Boming Zuo, Tianci Zeng, Jiankun Lu, Yuankai Wang, Boyang Liu, Hongbo Guo

**Affiliations:** ^1^ Department of Neurosurgery Center The National Key Clinical Specialty The Engineering Technology Research Center of Education Ministry of China on Diagnosis and Treatment of Cerebrovascular Disease Guangdong Provincial Key Laboratory on Brain Function Repair and Regeneration The Neurosurgery Institute of Guangdong Province Zhujiang Hospital Southern Medical University Guangzhou 510282 China

**Keywords:** glioblastoma, intron retention, lactylation, LUC7L2, temozolomide resistance

## Abstract

Temozolomide (TMZ) resistance remains the major obstacle in the treatment of glioblastoma (GBM). Lactylation is a novel post‐translational modification that is involved in various tumors. However, whether lactylation plays a role in GBM TMZ resistance remains unclear. Here it is found that histone H3K9 lactylation (H3K9la) confers TMZ resistance in GBM via LUC7L2‐mediated intron 7 retention of MLH1. Mechanistically, lactylation is upregulated in recurrent GBM tissues and TMZ‐resistant cells, and is mainly concentrated in histone H3K9. Combined multi‐omics analysis, including CUT&Tag, SLAM‐seq, and RNA‐seq, reveals that H3K9 lactylation is significantly enriched in the LUC7L2 promoter and activates LUC7L2 transcription to promote its expression. LUC7L2 mediates intron 7 retention of MLH1 to reduce MLH1 expression, and thereby inhibit mismatch repair (MMR), ultimately leading to GBM TMZ resistance. Of note, it is identified that a clinical anti‐epileptic drug, stiripentol, which can cross the blood–brain barrier and inhibit lactate dehydrogenase A/B (LDHA/B) activity, acts as a lactylation inhibitor and renders GBM cells more sensitive to TMZ in vitro and in vivo. These findings not only shed light on the mechanism of lactylation in GBM TMZ resistance but also provide a potential combined therapeutic strategy for clinical GBM treatment.

## Graphical abstract

Lactylation is upregulated in recurrent glioblastoma (GBM) tissues and temozolomide (TMZ)‐resistant cells, mainly concentrated in histone H3K9. H3K9 lactylation activates LUC7L2 transcription. LUC7L2 mediates MLH1 intron 7 retention to reduce MLH1 expression, thereby inhibit mismatch repair (MMR), ultimately leading to TMZ resistance. Targeting lactylation with stiripentol to enhance TMZ sensitivity is a promising combined therapeutic strategy for clinical GBM treatment.

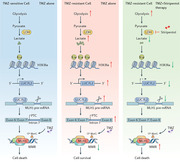

## 1 Introduction

Glioblastoma (GBM) is the most common malignant tumor of the central nervous system, with high mortality and recurrence rates.^[^ ^1^ ^]^ Surgical resection combined with chemoradiotherapy is the current conventional treatment; however, the prognosis remains very poor. The median survival time is ≈15 months, and the five‐year overall survival rate is 5%.^[^ ^2^ ^]^ Temozolomide (TMZ), a widely used oral chemotherapeutic drug, is the first‐line therapy in patients with GBM. Although TMZ treatment has therapeutic potential for patients in the early disease stage, long‐term use can lead to reduced sensitivity and even drug resistance.^[^ ^3^ ^]^ Therefore, improving the chemosensitivity of TMZ is a primary concern of clinicians and needs to be urgently addressed in clinical practice.

Lactylation is a novel type of post‐translational modification found on histones,^[^ ^4^ ^]^ which is an important way for lactate, an intermediate product of cellular metabolism, to exert biological functions.^[^ ^5^ ^]^ According to recent reports, histone lactylation plays a role in the regulation of the incidence and development of malignant tumors. Histone lactylation can promote the occurrence of ocular melanoma by upregulating the expression of m^6^A‐modified reader protein YTHDF2.^[^ ^6^ ^]^ Histone lactylation promotes PDGFR‐β to form a positive feedback loop to drive the aggressive development of clear cell renal cell carcinoma.^[^ ^7^ ^]^ Notably, histone lactylation also regulates tumor chemosensitivity. Histone lactylation increases bevacizumab resistance in colorectal cancer via promoting the expression of the autophagy enhancer protein RUBCNL.^[^ ^8^ ^]^ In addition, lactylation in GBM is associated with self‐renewal of stem cells, CAR‐T therapy and vasculogenic mimicry.^[^ ^9^ ^]^ However, whether lactylation plays a role in GBM TMZ resistance has not yet been reported, and further in‐depth investigation is urgently needed.

LUC7L2 is a highly conserved and widely expressed RNA‐binding protein that acts as a splicing factor and participates in disease occurrence and progression by mediating alternative splicing.^[^ ^10^ ^]^ It has been confirmed that LUC7L2 is closely related to the malignant progression of tumors. LUC7L2 is involved in chordoma cell survival through alternative splicing and is a potential treatment target.^[^ ^11^ ^]^ In myeloid neoplasms, LUC7L2 deficiency results in the significant upregulation of the expression of multiple spliceosomal factors and possibly contributes to pathogenesis.^[^ ^12^ ^]^ LUC7L2 is involved in the regulation of immune infiltration in malignant mesothelioma through alternative splicing.^[^ ^13^ ^]^ However, whether LUC7L2 participates in GBM TMZ resistance has not been reported, and further study is crucially required.

In this study, we investigated the functional role of lactylation in GBM TMZ resistance by using combined genome‐wide CUT&Tag, SLAM‐seq, and RNA‐seq to explore downstream regulatory mechanisms, and developed a combined therapeutic strategy against GBM.

## 2 Results

### 2.1 Histone H3K9 Lactylation has Increased Expression in Recurrent Glioblastoma, and Chronic TMZ Exposure Increases the Level of H3K9 Lactylation

**Figure 1:**
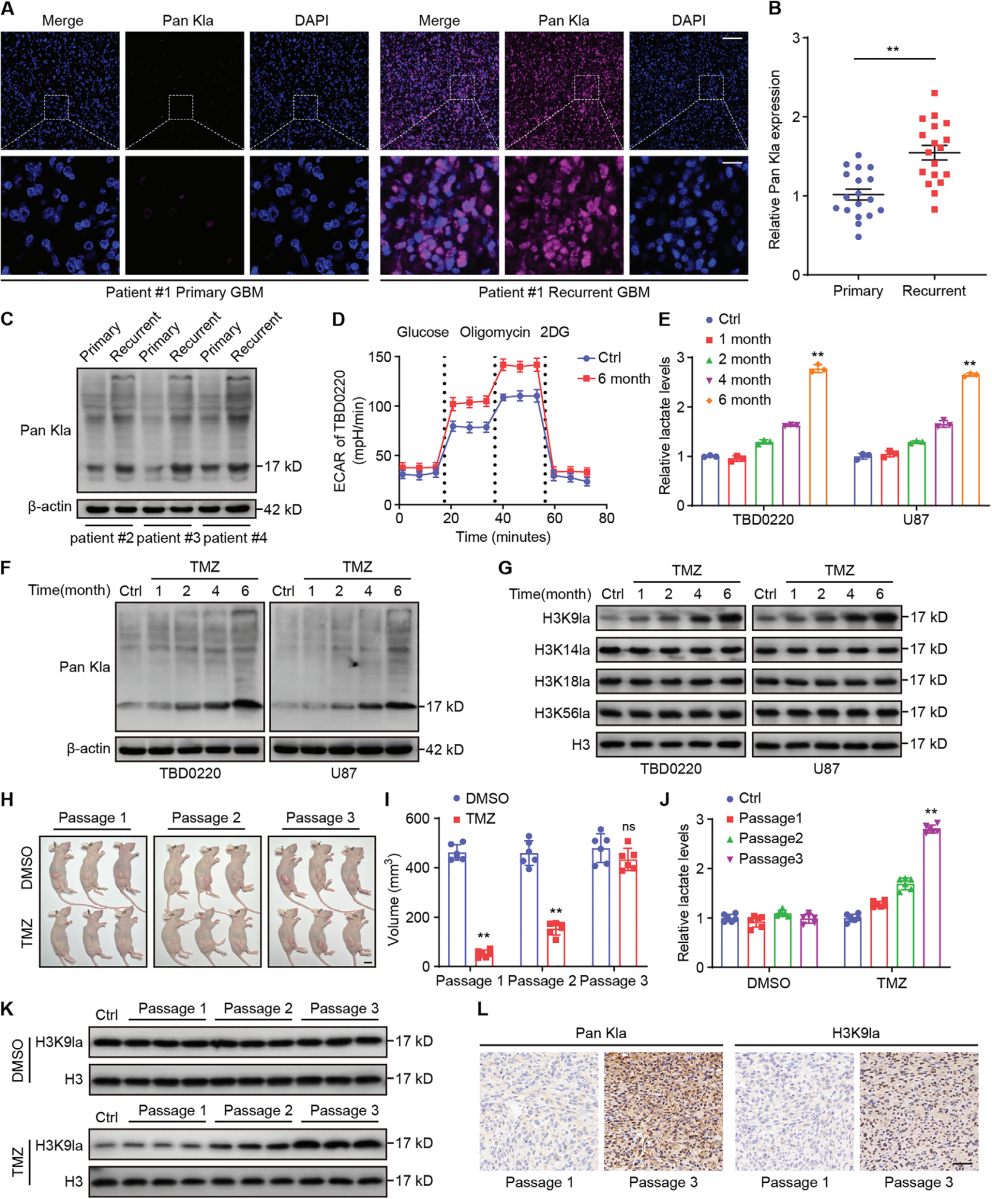
H3K9la is highly expressed in recurrent GBM, and chronic TMZ exposure increases H3K9la. A) Representative IF staining images of pan Kla in primary and recurrent GBM. Scale bar, top panel, 100 µm; bottom panel, 20 µm. B) Statistical results of pan Kla in primary and recurrent GBM. C) Pan Kla was measured by WB in primary and recurrent GBM. D) ECAR was measured in TBD0220 at the indicated time. E) Lactate levels were measured in TBD0220 and U87 at the indicated time compared to the control group. F) Pan Kla was detected by WB in TBD0220 and U87 at the indicated time compared to the control group. G) H3K9la, H3K14la, H3K18la, and H3K56la were measured by WB in TBD0220 and U87 at the indicated time. H) Oral gavage therapy with TMZ or DMSO was administered to mice that had received three subcutaneous implants of TBD0220 cells. Scale bar, 10 mm. I) The volume of tumors in various groupings and passages. J) The lactate levels of different groups and passages. K) WB analysis of H3K9la of different groups and passages. L) Representative IHC staining images of pan Kla and H3K9la in different groups and passages. Scale bar, 50 µm. All values are shown as the mean ± SD. ^**^ *p* < 0.01; ns: not significant.

To explore the role of lactylation in GBM TMZ resistance, we examined global lactylation in 18 pairs of primary and matched recurrent GBM tissues. Immunofluorescence staining showed noticeably higher levels of global lactylation in recurrent GBM than in primary tumors (**Figure** **1**A,B). Consistent herewith, WB assays showed that global lactylation levels were elevated in recurrent GBM, and a predominant band appeared ≈17 kDa, which may represent histone H3 (Figure 1C). To further explore the role of lactylation in TMZ resistance, TBD0220 and U87 GBM cells were exposed to TMZ. In clinical practice, GBM patients frequently need multiple, long‐term TMZ treatments. To imitate long‐term TMZ treatment in a clinical situation, the GBM cells were chronically treated with TMZ in vivo and in vitro by time and passage. Seahorse XF glycolytic stress assays were performed to identify the ECAR and found that chronic TMZ exposure remarkably increased the basal glycolytic rate (Figure 1D; Figure S1A, Supporting Information). Moreover, chronic TMZ exposure increased lactate and lactylation levels compared to control group (Figure 1E,F). Interestingly, as observed previously, a dominant band appeared near 17 kD. We examined various lactylation sites on histone H3 and found that the level of H3K9 lactylation (H3K9la) gradually increased with TMZ exposure (Figure 1G). Short‐term TMZ treatment did not affect the H3K9la level (Figure S1B, Supporting Information). Similar outcomes in a subcutaneous xenograft trial were observed; chronic TMZ exposure increased lactate and H3K9la levels (Figure 1H–K). IHC staining revealed elevated pan Kla and H3K9la levels after chronic TMZ exposure (Figure 1L). These data indicated that recurrent GBM is characterized by an elevated H3K9la level, which is likely to be involved in the TMZ resistance of GBM.

### 2.2 Inhibition of H3K9la Enhances GBM TMZ Sensitivity

**Figure 2:**
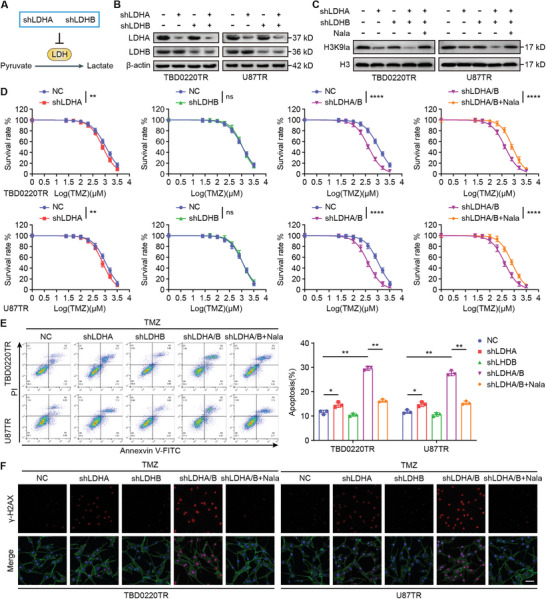
Inhibition of H3K9la enhances GBM TMZ sensitivity. A) Schematic representation of the target lactylation inhibition approaches. B) LDHA and LDHB levels were detected by WB after LDHA and LDHB knockdown. C) H3K9la levels were detected by WB after LDHA and LDHB knockdown and Nala addition. D) IC_50_ was evaluated after TMZ (200 µM). E) The apoptosis rate was measured by flow cytometry after TMZ (200 µM) treatment. F) Confocal IF of γ‐H2AX of TBD0220TR and U87TR after LDHA and LDHB knockdown and Nala addition. Scale bar, 50 µm. All values are shown as the mean ± SD. ^*^ *p* < 0.05, ^**^ *p* < 0.01, ^****^ *p* < 0.0001.

To evaluate whether H3K9la inhibition would sensitize GBM cells to TMZ, we reduced H3K9la levels in TMZ‐resistant (TR) cells, using shRNAs targeting LDHA and LDHB to attenuate lactate and H3K9la levels (**Figure** **2**A). Notably, LDHB deficiency had no effect on H3K9la levels, and LDHA deficiency inhibited H3K9la expression. However, simultaneous knockdown of LDHA and LDHB significantly suppressed H3K9la levels to a greater extent (Figure 2B,C), as well as lactate production (Figure S2A, Supporting Information). The addition of sodium lactate (Nala) in LDHA/B‐deficient TBD0220TR and U87TR cells successfully restored the H3K9la levels (Figure 2B,C). Further, knockdown of both LDHA and LDHB significantly reduced the IC_50_ of TMZ in TBD0220TR and U87TR cells, and this reduction was reverted upon the addition of Nala (Figure 2D; Table S1, Supporting Information). The results of colony formation experiments showed that knocking down both LDHA and LDHB markedly reduced the number of cell colonies, and Nala addition reversed this result (Figure S2B, Supporting Information). Knockdown of LDHA/B substantially increased the apoptotic rate, whereas the addition of Nala reduced the apoptotic rate (Figure 2E). Confocal immunofluorescence analysis revealed that knockdown of LDHA/B greatly increased the expression of γ‐H2AX, whereas Nala addition decreased the γ‐H2AX levels (Figure 2F). The above results indicated that inhibition of H3K9la enhances the sensitivity of GBM to TMZ.

### 2.3 Stiripentol Enhances the Efficacy of TMZ by Inhibiting H3K9la

**Figure 3:**
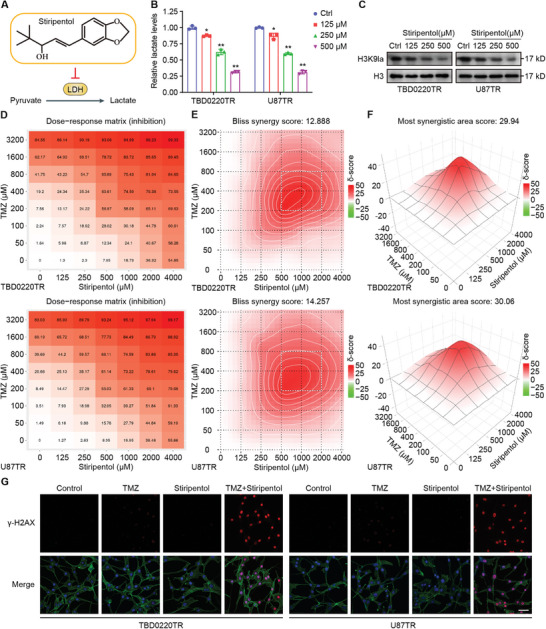
Stiripentol enhances the efficacy of TMZ by inhibiting H3K9la. A) Schematic diagram of stiripentol inhibiting LDHA/B. B) Lactate levels were measured after stiripentol treatment. C) H3K9la levels were detected by WB after stiripentol treatment. D) Combination dose‐response matrices of cell inhibition by stiripentol and TMZ treatment. E) Bliss synergy score by stiripentol and TMZ treatment. F) Most synergy area score by stiripentol and TMZ treatment. G) Confocal IF of γ‐H2AX of TBD0220TR and U87TR after stiripentol (500 µM) and TMZ (200 µM) treatment. Scale bar, 50 µm. All values are shown as the mean ± SD. ^*^ *p* < 0.05, ^**^ *p* < 0.01.

Stiripentol, an anti‐epileptic drug used in clinical treatment, was found to cross the blood–brain barrier and inhibit cerebral LDHA/B activity^[^ ^14^ ^]^ (**Figure** **3**A).We treated TBD0220TR and U87TR cells with stiripentol and found that the lactate levels in the cells gradually decreased with increasing concentrations of stiripentol (Figure 3B). Similarly, H3K9la levels decreased with increasing concentrations of stiripentol (Figure 3C). To investigate whether stiripentol and TMZ exert synergistic effects, we treated the cells with TMZ or stiripentol alone or in combination. According to the concentration gradient and the corresponding inhibition index, combination dose‐response matrices were generated (Figure 3D). The Bliss synergy scores were calculated and the results showed that the average (and maximum) scores of the antitumor response attributable to the drug interaction were 12.888 (29.94) in TBD0220TR cells and 14.257 (30.06) in U87TR cells (Figure 3E,F). In general, combination dose‐response matrices and Bliss synergy scores revealed a synergistic effect of the TMZ and stiripentol combination regimen in these cells (Bliss synergy scores >10). Stiripentol showed the best synergistic effect at 500 µM; therefore, we selected this concentration for use in subsequent experiments. The results of colony formation assays showed that, compared with TMZ alone, TMZ + stiripentol more strongly inhibited colony formation (Figure S3A, Supporting Information). The results of apoptosis assays showed that the combination regimen induced a significantly elevated apoptotic rate compared to TMZ alone (Figure S3B, Supporting Information). Confocal immunofluorescence results showed that, compared with that in the TMZ group, γ‐H2AX expression was considerably elevated in the combination treatment group (Figure 3G). Taken together, these results revealed that stiripentol greatly enhances the effect of TMZ on GBM cells by inhibiting H3K9la.

### 2.4 H3K9la Activates *LUC7L2* Transcription

**Figure 4:**
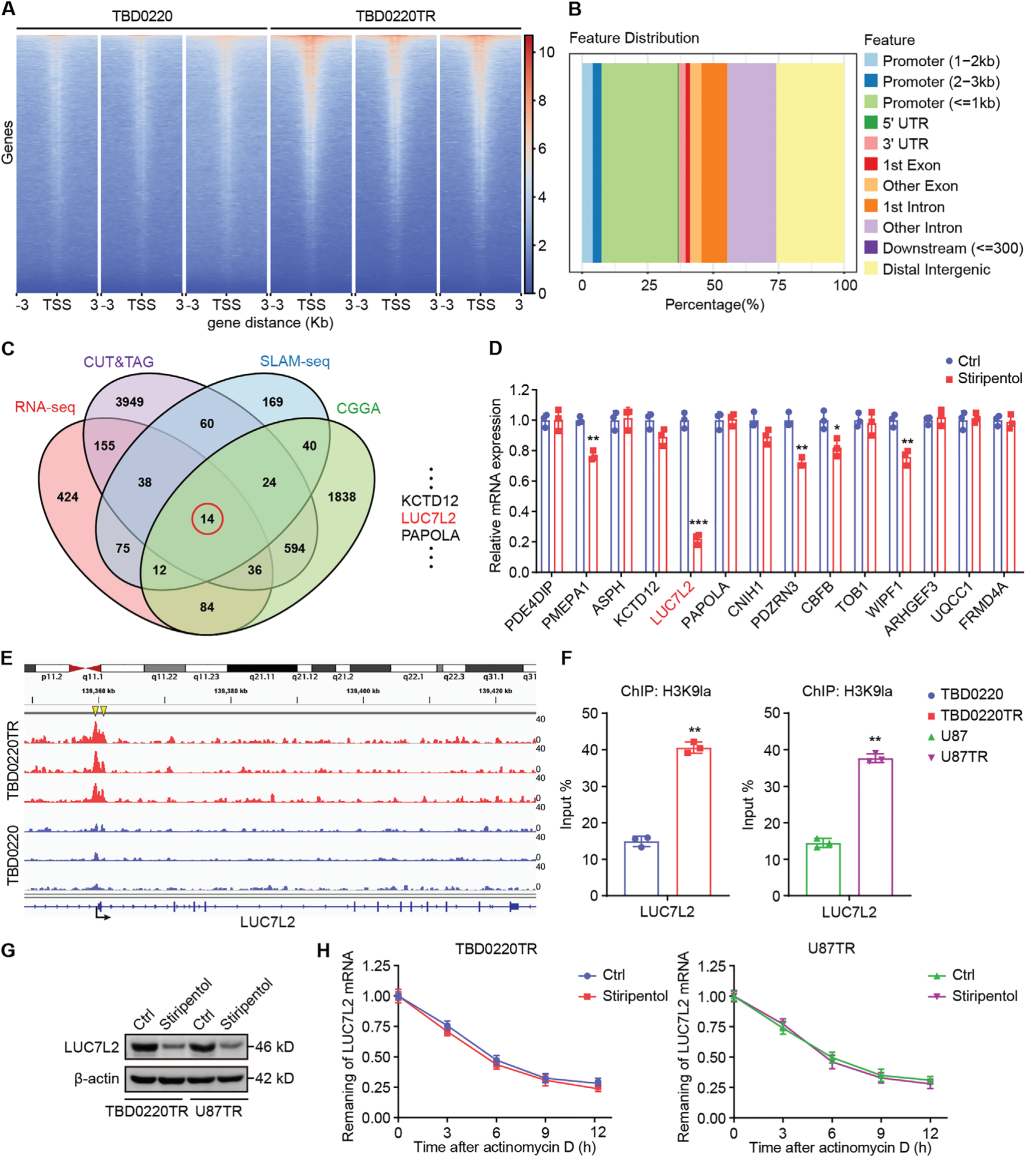
H3K9la activates LUC7L2 transcription. A) The heatmap illustrates the CUT&Tag tag counts on the various H3K9la enrichment peaks in TBD0220 and TBD0220TR cells. B) Genome‐wide distribution of upregulated H3K9la enrichment peaks in TBD0220TR cells. C) Venn Diagram of CUT&Tag, SLAM‐seq, RNA‐seq, and CGGA. D) mRNA levels were measured by qPCR after stiripentol (500 µM) treatment. E) CUT&Tag signal traces from genome browsers at sample target gene locations. The H3K9la peak areas on the LUC7L2 promoters are shown by the yellow rectangles. F) Using antibodies against H3K9la, qChIP analysis of the identified promoters was conducted in parent cells and TMZ‐resistant cells. G) LUC7L2 levels were detected by WB after stiripentol (500 µM) treatment. H) Effects of stiripentol (500 µM) on LUC7L2 mRNA stability in Act D‐treated cells (5 µg mL^−1^). All values are shown as the mean ± SD. ^*^ *p* < 0.05, ^**^ *p* < 0.01, ^***^ *p* < 0.001.

Histone lactylation affects the transcriptional activation of target genes.^[^ ^4^ ^]^ To explore the role of H3K9la in GBM TMZ resistance, a genome‐wide CUT&Tag^[^ ^15^ ^]^ analysis was performed to identify potential genes regulated by H3K9la in TMZ‐resistant cells. Briefly, CUT&Tag using antibodies against H3K9la and data analysis using deep‐Tools revealed obvious enrichment of H3K9la in TBD0220TR cells compared to TBD0220 cells (**Figure** **4**A). The comparison revealed differential H3K9la‐binding peaks in TBD0220TR cells, and 36.15% of the peaks were located within promoter sequences (≤3 Kb; Figure 4B; Figure S4A, Supporting Information). To identify target genes of H3K9la transcriptional activation, we combined CUT&Tag with SLAM‐seq, a method for the direct quantification of newly synthesized mRNAs.^[^ ^16^ ^]^ The above sequencing results were combined with RNA‐seq and Chinese Glioma Genome Atlas data (CGGA, primary vs recurrent) for analysis, focusing on 14 target genes (Figure 4C). In TBD0220TR cells treated with stiripentol, we found that *LUC7L2* mRNA expression was significantly reduced. Therefore, we focused on this target gene in subsequent experiments (Figure 4D). To confirm that H3K9la activates LUC7L2 transcription, we first evaluated H3K9la signals in this gene. We perceived a substantially enrichment of the H3K9la signal in the *LUC7L2* promoter by using Integrative Genomics Viewer (Figure 4E). Quantitative chromatin immunoprecipitation (ChIP‐qPCR) analysis revealed that the H3K9la level in the *LUC7L2* promoter was greatly elevated in TMZ‐resistant cells compared to parent cells (Figure 4F; Figure S4B, Supporting Information). In addition, the ChIP‐qPCR assays showed that the level of binding of EP300, a histone lactylation writer, to the *LUC7L2* promoter was significantly elevated in TMZ‐resistant cells compared to parent cells (Figure S4C, Supporting Information). After treatment with stiripentol, LUC7L2 expression was significantly decreased at both the mRNA (Figure S4D, Supporting Information) and protein (Figure 4G) levels. Furthermore, mRNA stability experiments suggested that stiripentol did not affect the mRNA stability of *LUC7L2* (Figure 4H). Taken together, these results indicated that H3K9la is positively regulating LUC7L2 transcription.

### 2.5 LUC7L2 Promotes GBM TMZ Resistance, and Overexpression of LUC7L2 Reverses the Effect of Stiripentol

**Figure 5:**
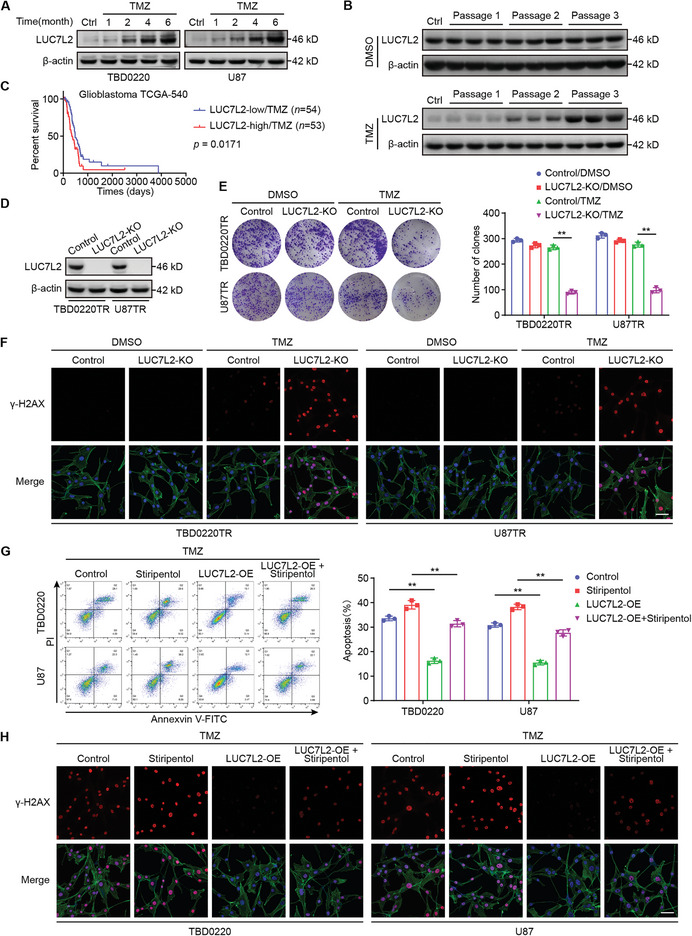
LUC7L2 promotes TMZ resistance in GBM, and overexpression of LUC7L2 reverses the effect of stiripentol. A) LUC7L2 levels were measured by WB in the chronic TMZ exposure model of TBD0220 and U87 cells at the indicated time. B) WB analysis of LUC7L2 of different groups and passages in chronic TMZ exposure model of mice. C) Using data from the GBM TCGA‐540 database, Kaplan–Meier graphs highlight the overall survival of GBM patients with high or low LUC7L2 expression undergoing TMZ treatment. D) LUC7L2 levels were measured by WB after LUC7L2 knockout. E) Colony formation assays were performed after LUC7L2 knockout. F) Confocal IF of γ‐H2AX of TBD0220TR and U87TR after LUC7L2 knockout. Scale bar, 50 µm. G) The apoptosis rate was measured by flow cytometry with or without LUC7L2 overexpression in cells treated with stiripentol (500 µM). H) Confocal IF of γ‐H2AX of TBD0220TR and U87TR with or without LUC7L2 overexpression in cells treated with stiripentol (500 µM). Scale bar, 50 µm. All values are shown as the mean ± SD. ^**^ *p* < 0.01.

To investigate the role of LUC7L2 in GBM TMZ resistance, we first detected LUC7L2 expression in in vitro and in vivo chronic TMZ exposure models. We found that LUC7L2 mRNA and protein expression increased with chronic TMZ exposure (**Figure** **5**A,B; Figure S5A,B, Supporting Information). Data from The Cancer Genome Atlas (TCGA) and CGGA indicated that high LUC7L2 levels were associated with a poor prognosis (Figure 5C; Figure S5C, Supporting Information). We generated *LUC7L2*‐knockout (KO) TBD0220TR and U87TR cells (Figure 5D) and evaluated the effect of *LUC7L2* KO on the IC_50_ of TMZ. The results showed that *LUC7L2* KO significantly reduced the IC_50_ of TMZ (Figure S5D and Table S1, Supporting Information). Cell colony formation experiments revealed that *LUC7L2* KO enhanced the inhibitory effect of TMZ on colony formation (Figure 5E). Further, *LUC7L2* KO substantially elevated the apoptotic rate induced by TMZ (Figure S5E, Supporting Information). Confocal immunofluorescence results showed that, compared with that in the TMZ group, γ‐H2AX expression was significantly elevated in the *LUC7L2*‐KO/TMZ group (Figure 5F). Taken together, these results revealed that LCU7L2 deficiency greatly enhances the effect of TMZ on GBM cells.

As we had found that LUC7L2 expression is regulated by H3K9la, we next aimed to determine whether the sensitization to TMZ by stiripentol could be suppressed by a gain of LUC7L2. After overexpressing LUC7L2 in TBD0220 and U87 cells, the sensitization to TMZ by stiripentol was partially compromised, as indicated by the increased IC_50_ of TMZ (Figure S5F and Table S1, Supporting Information), increased colony formation (Figure S5G, Supporting Information), and suppressed apoptosis (Figure 5G). γ‐H2AX expression was partially compromised in the *LUC7L2*‐OE + stiripentol group compared to the stiripentol group (Figure 5H). In addition, overexpression of LUC7L2 in TBD0220 and U87 cells enhanced TMZ resistance. Collectively, these results implied that H3K9la promotes TMZ resistance through LUC7L2.

### 2.6 LUC7L2 Promotes TMZ Resistance by Inhibiting MLH1 Expression

**Figure 6:**
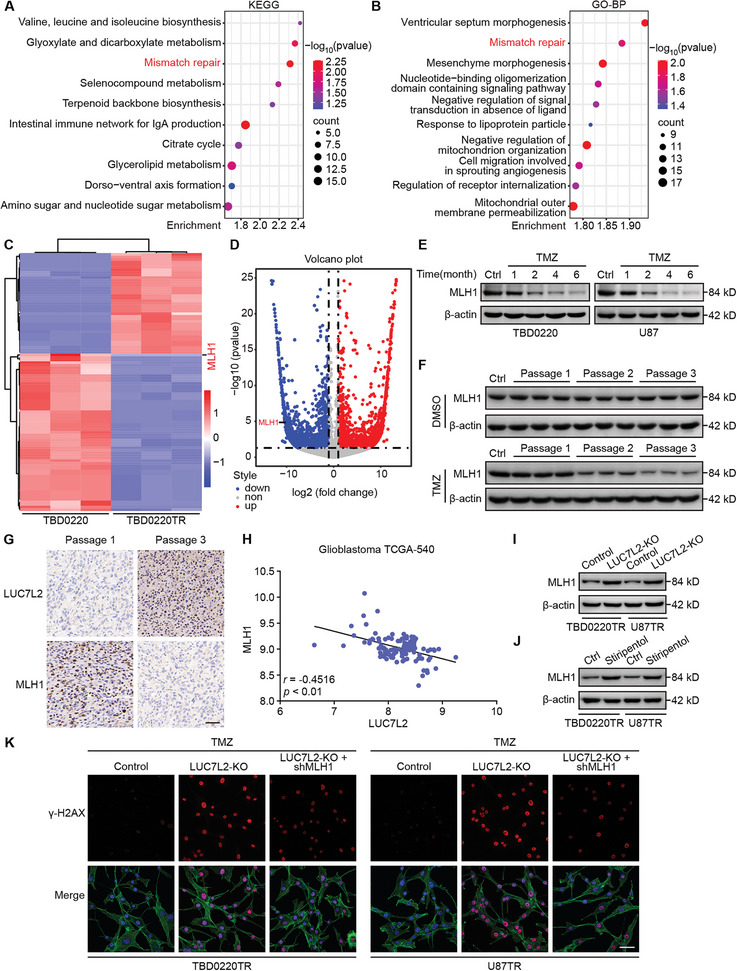
LUC7L2 promotes TMZ resistance by inhibiting MLH1 expression. A) KEGG analysis of RNA‐seq. B) GO‐BP analysis of RNA‐seq. C) Clustering heatmap of mRNA from RNA‐seq. D) Volcano plot of mRNA from RNA‐seq. E) MLH1 levels were measured by WB in a chronic TMZ exposure model of TBD0220 and U87 cells at the indicated time. F) WB analysis of MLH1 of different groups and passages in a chronic TMZ exposure mouse model. G) Representative IHC staining images of LUC7L2 and MLH1 in different groups and passages. H) The relationship between LUC7L2 and MLH1 in the TCGA‐540 database. I) MLH1 levels were measured by WB after LUC7L2 knockout. J) MLH1 levels were detected by WB after stiripentol (500 µM) treatment. K) Confocal IF of γ‐H2AX of TBD0220TR and U87TR with LUC7L2 knockout and MLH1 knockdown. Scale bar, 50 µm.

To explore the downstream targets of the splicing factor LUC7L2, we performed KEGG and GO‐BP analyses and found that mismatch repair (MMR) was significantly differentially enriched in TBD0220TR cells (**Figure** **6**A,B). Clustering analysis and a volcano plot revealed that MLH1, a key factor of MMR, was significantly downregulated in TMZ‐resistant cells (Figure 6C,D), and MLH1 is not a H3K9la transcriptionally activated gene (Figure S6A, Supporting Information). One major mechanism that leads to TMZ resistance in GBM is the absence of the MMR system and MLH1 deficiency can confer GBM TMZ resistance.^[^ ^17^ ^]^ ChIP‐qPCR assay results revealed no markedly difference of H3K9la in the *MLH1* promoter in the TMZ‐resistant cells compared to parental cells (Figure S6B, Supporting Information). This indicated that LUC7L2 may regulate MLH1 expression at the post‐transcriptional level. To investigate the role of MLH1 in GBM TMZ resistance, we first detected MLH1 expression in in vitro and in vivo chronic TMZ exposure models. We found that MLH1 expression decreased with chronic TMZ exposure (Figure 6E,F). IHC staining showed that chronic TMZ exposure increased the expression of LUC7L2 and decreased that of MLH1 (Figure 6G). Data from TCGA revealed a significant negative correlation between LUC7L2 and MLH1 expression (Figure 6H). Next, we examined the effect of LUC7L2 deficiency on MLH1 expression. WB and qPCR results showed that LUC7L2 deficiency strongly increased MLH1 expression (Figure 6I; Figure S6C, Supporting Information). Similarly, stiripentol markedly increased the expression of MLH1 (Figure 6J). Next, we investigated whether knockdown of MLH1 would alter the effect of LUC7L2 deficiency. MLH1 deficiency partially restored the IC_50_ of TMZ (Figure S6D and Table S1, Supporting Information) and the colony formation (Figure S6E, Supporting Information) and apoptosis rate (Figure S6F, Supporting Information) in LUC7L2‐deficient cells. Moreover, γ‐H2AX expression was partially compromised in the *LUC7L2*‐KO + shMLH1 group compared to the *LUC7L2*‐KO group (Figure 6K). Collectively, these data suggested that the LUC7L2/MLH1 axis contributes to TMZ resistance in GBM.

### 2.7 LUC7L2 Regulates *MLH1* Intron 7 Retention

**Figure 7:**
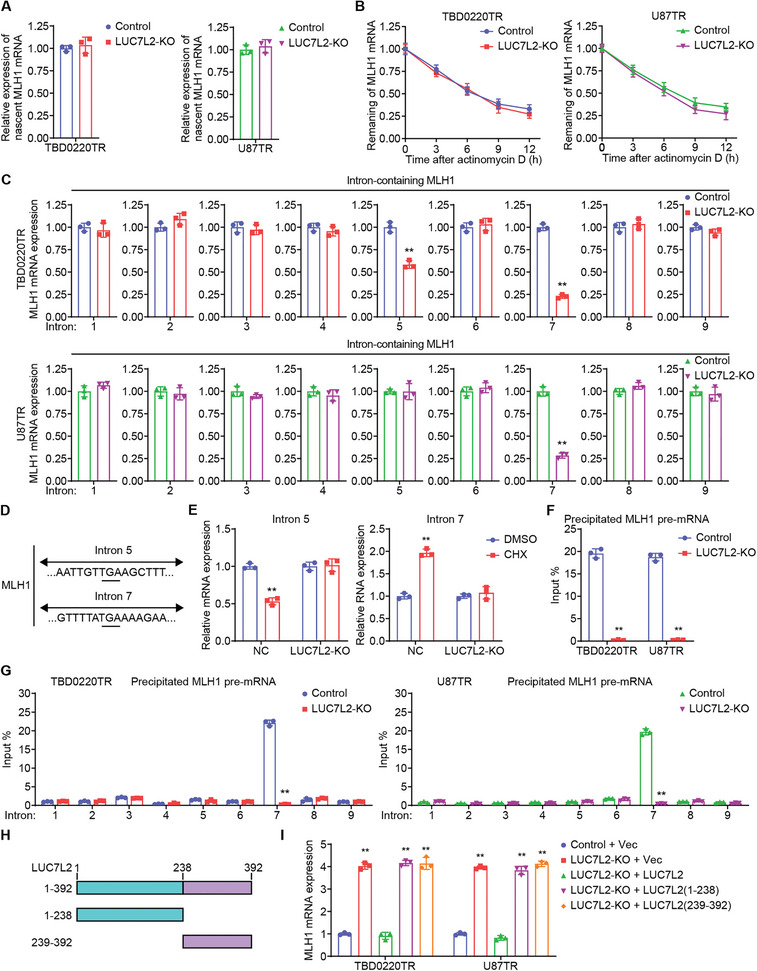
LUC7L2 regulates MLH1 intron 7 retention. A) Nascent MLH1 mRNA levels were measured after LUC7L2‐KO. B) Influence of LUC7L2‐KO on the stability of MLH1 mRNA in TBD0220TR and U87TR cells treated with Act D (5 µg mL^−^^1^). C) Influence of LUC7L2‐KO on MLH1 transcript levels with retained introns. D) *MLH1* partial sequences displaying the PTCs in introns 5 and 7. E) Effects of LUC7L2‐KO on the alteration in MLH1 transcripts with retained introns caused by CHX (30 µg mL^−1^). F) RIP assays were performed with LUC7L2 antibody. G) CLIP assays were performed with LUC7L2 antibody, and cells were treated with UV (254 nm, 400 mJ cm^−2^). H) LUC7L2 and its truncation mutants are shown schematically. I) Influence of LUC7L2 reconstitution and truncation mutants on MLH1. All values are shown as the mean ± SD. ^**^ *p* < 0.01.

To investigate the specific mechanism by which LUC7L2 inhibits MLH1 expression, we examined *MLH1* nascent mRNA expression. The results showed that there was no significant difference in the nascent *MLH1* transcript levels between the LUC7L2‐deficient group and the control group (**Figure** **7**A), indicating that *MLH1* transcription is not regulated by LUC7L2. Subsequently, we evaluated the half‐life of MLH1 mRNA and discovered that there was no discernible change between the LUC7L2‐deficient and control groups (Figure 7B). These findings showed that MLH1 mRNA stability is not regulated by LUC7L2. Collectively, these data implied that a step of MLH1 pre‐mRNA splicing is regulated by LUC7L2. Next, using primers that targeted each individual intron and the exon next to it, we performed qPCR to identify the expression of MLH1 transcripts with unspliced introns in TBD0220TR and U87TR cells. The levels of MLH1 transcripts including intron 7 were found to be lower in LUC7L2‐deficient TBD0220TR and U87TR cells, yet those containing intron 5 were found to be lower solely in TBD0220TR cells. (Figure 7C; Figure S7A, Supporting Information). Sequence analysis results suggested that introns 5 and 7 of *MLH1* comprise premature termination codons (PTCs; Figure 7D), which would result in rapid transcript degradation via the nonsense‐mediated decay (NMD) pathway. Treatment with cycloheximide, an NMD pathway inhibitor, enhanced the levels of intron 7‐containing *MLH1* transcripts, but not intron 5‐containing *MLH1* transcripts (Figure 7E), indicating that MLH1 intron 7 retention accelerates their degradation via the NMD pathway. Nevertheless, in LUC7L2‐deficient cells, cycloheximide had no effect on MLH1 transcripts that included intron 7. RIP assays revealed that *MLH1* pre‐mRNA can bind to LUC7L2 (Figure 7F). Next, the binding site of LUC7L2 to the *MLH1* transcript was then carefully mapped using CLIP assays. The findings demonstrated that LUC7L2 bound to *MLH1* intron 7 (Figure 7G; Figure S7B, Supporting Information). Furthermore, we evaluated whether *MLH1* intron retention by LUC7L2 is reliant on its RNA‐binding capacity, which is linked to its C‐terminal Arg/Ser‐rich region.^[^ ^18^ ^]^ We performed experiments of reconstitution and discovered that wild‐type LUC7L2 suppressed the expression of MLH1 but not its N‐ or C‐terminal truncation mutants. (Figure 7H,I), indicating that intact LUC7L2 is important for inhibiting MLH1 expression. Collectively, these data revealed that LUC7L2 favors the retention of intron 7 in *MLH1* transcripts, resulting in NMD‐dependent degradation and suppression of MLH1 expression.

### 2.8 Stiripentol Increases TMZ Sensitivity In Vivo

**Figure 8:**
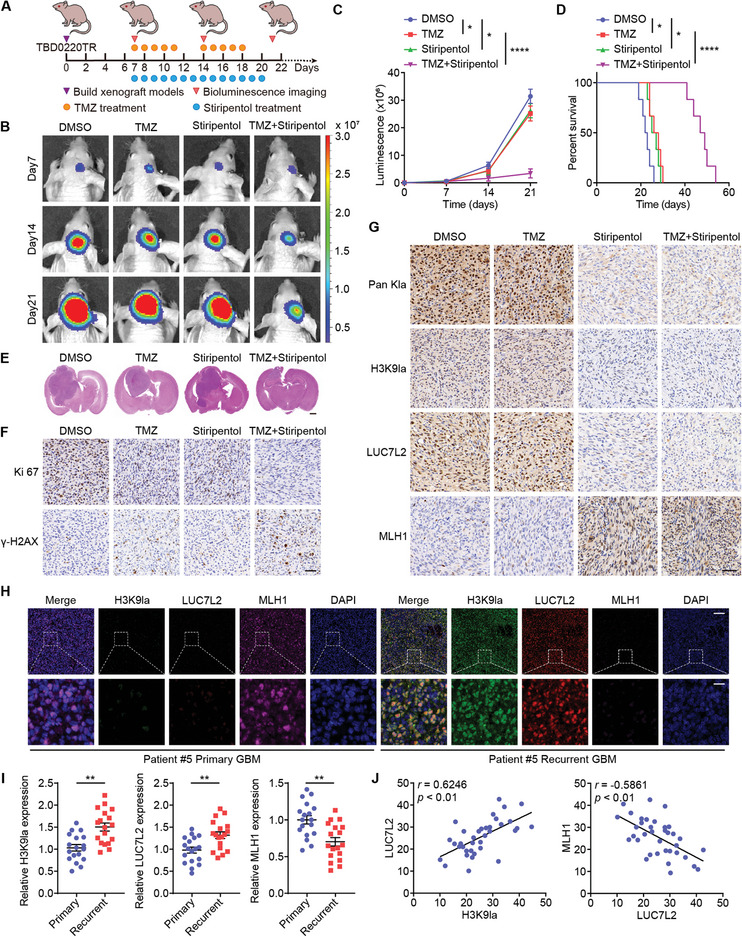
Stiripentol increases TMZ sensitivity in vivo. A) Tumor‐bearing nude mice were treated with DMSO, TMZ (5 mg kg^−1^), stiripentol (150 mg kg^−1^), or TMZ (5 mg kg^−1^) + stiripentol (150 mg kg^−1^) by gavage. B) Images showing bioluminescence from representative mice across all groups. C) The intensity of each group's bioluminescence was measured. D) Kaplan–Meier survival curves were evaluated in nude mice. E) Representative H&E images of all nude mice groups. Scale bar, 1 mm. F) Ki‐67 and γ‐H2AX expression of representative IHC of nude mice tumor tissues. Scale bar, 50 µm. G) IHC of tumor tissues showing pan Kla, H3K9la, LUC7L2, and MLH1 expression. Scale bar, 50 µm. H) IF staining images of H3K9la, LUC7L2, and MLH1 in primary and recurrent GBM tissues. Scale bar: top panel, 100 µm; bottom panel, 20 µm. I) Statistical results of H3K9la, LUC7L2, and MLH1 in 18 paired primary and recurrent GBM tissues. J) The relationship between H3K9la, LUC7L2, and MLH1. All values are shown as the mean ± SD. ^*^ *p* < 0.05, ^**^ *p* < 0.01, ^****^ *p* < 0.0001.

To investigate whether stiripentol can synergize with TMZ in vivo, TBD0220TR cells were utilized to generate an orthotopic GBM model (**Figure** **8**A). Bioluminescence imaging revealed a minor inhibition of tumor development by both TMZ and stiripentol. However, animals receiving the combined treatment showed the least amount of tumor growth (Figure 8B,C) and the longest median survival time (Figure 8D). Sections of brain tumors stained with H&E showed that tumor size was the smallest in the TMZ + stiripentol group among all groups (Figure 8E). IHC stain results revealed that Ki67 levels were significantly reduced in the TMZ + stiripentol group compared to the TMZ group (Figure 8F). Furthermore, we found that TMZ therapy induced a low level of γ‐H2AX expression, whereas the combined regime significantly increased γ‐H2AX levels. Moreover, in brain sections of mice of the DMSO and TMZ‐alone groups, we observed high levels of pan Kla, H3K9la, LUC7L2, and low level of MLH1, whereas this trend was reversed in the stiripentol and TMZ + stiripentol groups (Figure 8G). Collectively, these findings suggested that stiripentol substantially enhanced TMZ sensitivity in vivo.

To assess the biosafety of TMZ + stiripentol combination therapy, we examined the body weights of the mice after 4 weeks of therapies. We found no significant body weight changes in stiripentol‐treated mice compared to control mice. While TMZ treatment did induce a decrease in body weight, it was not worsened by the combination treatment (Figure S8A, Supporting Information). The mice were euthanized in the fourth week of therapy, and organ samples were taken out for H&E staining. There was no discernible target organ toxicity found (Figure S8B, Supporting Information). Further, routine blood and liver and kidney function tests revealed no organ toxicity (Tables S2 and S3, Supporting Information). We used immunofluorescence imaging to detect the expression of H3K9la, LUC7L2, and MLH1 in paired primary and recurrent GBM (Figure 8H). The data indicated that H3K9la and LUC7L2 were considerably higher in recurrent GBM than in primary GBM, whereas MLH1 was significantly downregulated in recurrent GBM (Figure 8I). Moreover, H3K9la was positively correlated with LUC7L2 expression, which was negatively correlated with MLH1 expression (Figure 8J).

**Figure 9:**
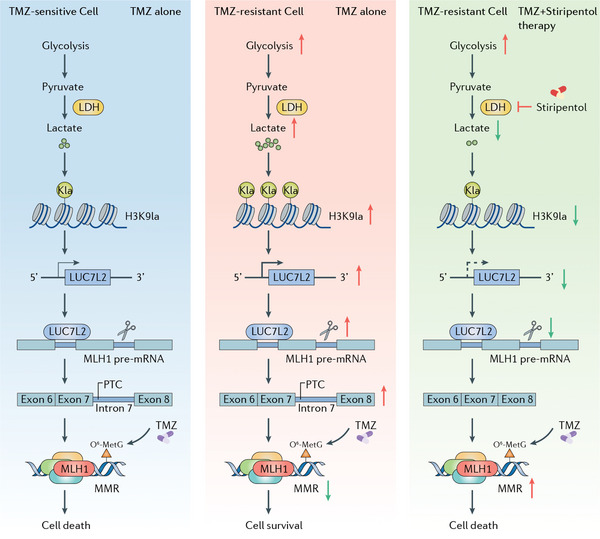
Schematic illustration depicting the mechanism of H3K9la promoting GBM TMZ resistance via LUC7L2‐mediated MLH1 intron 7 retention and a combined therapeutic strategy of TMZ and stiripentol for clinical GBM management.

In summary, this study demonstrated that lactylation levels are upregulated in recurrent GBM and TMZ‐resistant cells. Upregulated H3K9la activates *LUC7L2* transcription, and LUC7L2 mediates *MLH1* intron 7 retention to reduce MLH1 expression, inhibiting MMR pathway, and ultimately promoting GBM TMZ resistance. The anti‐epileptic drug stiripentol significantly enhances the sensitivity to TMZ by inhibiting lactylation (**Figure** **9**).

## 3 Discussion

Postoperative chemotherapy is the most effective strategy for the treatment of GBM. The median survival time can be extended with TMZ treatment. But as therapy progresses, GBM patients will gradually develop TMZ resistance.^[^ ^19^ ^]^ GBM occurrence and development is accompanied by metabolic reprogramming, such as glycolysis.^[^ ^20^ ^]^ This study revealed that in a chronic TMZ exposure model, the glycolysis of GBM cells gradually increased, and the metabolite lactate was continuously accumulated. This result suggested that active glycolysis and lactate accumulation are closely related to TMZ resistance in GBM. It has long been believed that lactate is a waste product of metabolism. Recent researches have demonstrated that lactate participates in a number of processes related to carcinogenesis and the advancement of cancer through lactylation.^[^ ^5^, ^21^ ^]^ Therefore, we measured lactylation levels in recurrent and primary GBM samples, as well as in TMZ‐resistant cells and parental cells. The results showed that lactylation was substantially increased in recurrent GBM and TMZ‐resistant cells, indicating a potential role for lactylation in the process underlying GBM TMZ resistance.

Lactylation was first reported to occur on histones, and numerous lactylation sites, such as H3K9la, H3K18la, H4K5la, and H4K12la, have been identified.^[^ ^22^ ^]^ Increased histone lactylation at gene promoters has been shown to induce gene expression via transcription activation.^[^ ^4^ ^]^ H3K18la facilitates the immunosuppression of tumor‐infiltrating myeloid cells by activating METTL3 transcription and mediating RNA m^6^A modification.^[^ ^23^ ^]^ Hepatocellular carcinoma tumorigenicity was decreased by blocking H3 histone lactylation at H3K9la and H3K14la locations.^[^ ^24^ ^]^ In the current investigation, we found that H3K9la levels were dramatically increased in recurrent GBM tissues and TMZ‐resistant cells, and inhibition of H3K9la enormously increased the sensitivity to TMZ. By combining data from genome‐wide CUT&Tag, SLAM‐seq, and RNA‐seq, we discovered that H3K9la strongly enriched in the *LUC7L2* promoter area and stimulated *LUC7L2* transcription. Moreover, overexpression of LUC7L2 partially reversed the inhibition of TMZ sensitization caused by suppression of H3K9la. Taken together, our findings indicated that H3K9la promotes TMZ resistance by activating LUC7L2 transcription.

The RNA‐binding protein LUC7L2 is a splicing factor that is involved in disease onset and progression through alternative RNA splicing, including intron retention.^[^ ^12^, ^25^ ^]^ In fact, considerable splicing changes, such as intron retention, are frequently seen in tumor cells and linked to the advancement and recurrence of cancer.^[^ ^26^ ^]^ It has been demonstrated that up to 80% of human protein‐coding genes are affected by intron retention.^[^ ^27^ ^]^ Moreover, intron retention is also exclusive to certain tissues and cell types. It is more common in immunological and brain cell types but less common in muscle and embryonic stem cells.^[^ ^28^ ^]^ When intron retention occurs in mature mRNA transcripts, they can be detected by monitoring mechanisms and degraded by NMD, as they usually contain in‐frame PTCs.^[^ ^29^ ^]^ Intron retention is strongly associated with drug resistance of cancer. DHX38 restricts chemoresistance by regulating *RELL2* intron retention in pancreatic ductal adenocarcinoma.^[^ ^30^ ^]^ The retention of intron 2 in *CD19* confers to CART‐19 resistance in leukemia.^[^ ^31^ ^]^ In our study, LUC7L2 was found to mediate *MLH1* intron 7 retention, and PTCs in intron 7 were recognized by NMD and led to *MLH1* mRNA degradation and thus, suppression of MLH1 expression. MLH1 deficiency partially reversed the effect of LUC7L2 deficiency on TMZ sensitivity. Therefore, LUC7L2 promotes GBM TMZ resistance by mediating *MLH1* intron 7 retention.

Stiripentol is a clinically used anti‐epileptic drug and is approved by the FDA for the therapy of Dravet syndrome. A preclinical investigation in mice showed that stiripentol can penetrate the blood–brain barrier and inhibit cerebral LDHA/B activity.^[^ ^14^ ^]^ We found that, in vitro, stiripentol significantly inhibited the expression of H3K9la and LUC7L2, but promoted that of MLH1. Moreover, stiripentol and TMZ exerted a strong synergistic effect. In in vivo experiments, stiripentol markedly inhibited the expression of H3K9la and LUC7L2, but promoted that of MLH1, and combination therapy with stiripentol and TMZ dramatically decreased tumor growth and substantially prolonged the survival of mice. In brief, stiripentol has an amazing sensitization effect on TMZ treatment in GBM.

There are certain limitations on this study that should be carefully considered. The clinical samples included in this study have to be further expanded due to the difficult availability of paired samples of primary and recurrent clinical GBM patients. Besides, there is a lack of protein post‐translational modification‐related databases for expression and survival assistant analysis. In addition, there is currently a lack of inhibitors targeting specific protein lactylation site, pending further studies in the future.

In summary, this study for the first time revealed that lactylation levels are high in recurrent GBM and TMZ‐resistant cells. Upregulated H3K9la activates *LUC7L2* transcription, and LUC7L2 mediates *MLH1* intron 7 retention to reduce MLH1 expression, inhibiting MMR, and ultimately promoting GBM TMZ resistance. The anti‐epileptic drug stiripentol significantly enhances the sensitivity to TMZ by inhibiting lactylation. This study provided a new perspective on the mechanism of TMZ resistance in GBM and paves the way to new combination therapies to improve the prognosis of GBM.

## 4 Experimental Section

### Clinical GBM Samples

GBM surgeries were conducted at the Neurosurgery Department, Zhujiang Hospital, Southern Medical University (Guangzhou, China), between January 2013 and July 2022. Eighteen patients were enrolled in the study, each contributing paired primary and recurrent GBM tissues. The samples underwent histological diagnosis at Zhujiang Hospital's Pathology Department, Southern Medical University, following the World Health Organization classification. All samples are isocitrate dehydrogenase wild‐type. All patients provided written informed consent. The research adhered to the Declaration of Helsinki and was approved by the Ethics Committee of Zhujiang Hospital, Southern Medical University.

### Cell Culture and Chemicals

The patient‐derived xenograft GBM primary cell line TBD0220 derived from a female GBM patient with no history of radiation therapy or chemotherapy and underwent surgery at Hebei University‐affiliated hospital,^[^ ^32^ ^]^ and GBM cell line U87 was procured from the cell bank of the Chinese Academy of Sciences. TMZ‐resistant cells (TBD0220TR and U87TR) were derived from TBD0220 and U87 cells. All cells were maintained at 37 °C with 100% humidity and 5% CO_2_. Briefly, TBD0220 and U87 cells were exposed to a concentration of TMZ that was gradually increased from 1.25 mmol L^−1^ to a maximal dose of 160 mmol L^−1^. During the first induction phase, the culture media containing TMZ was replaced every 72 h. The concentration of TMZ was increased after maintaining the initial dose for 14 days and observing consistent cell growth. At each incremental step, the cultures were maintained for three to four weeks. The overall procedure took half a year.^[^ ^33^ ^]^ To maintain authenticity, all GBM cell lines were cryopreserved in liquid nitrogen. TBD0220 and TBD0220TR cells were cultivated in DMEM/F12 (Gibco) with 10% FBS, whereas U87 and U87TR cells were cultured in DMEM (Gibco) with 10% FBS. TMZ (#HY‐17364) and stiripentol (#HY‐103392) were acquired from MedChemExpress (China). TMZ and stiripentol were dissolved in DMSO (Sigma‐Aldrich, D2650, USA) to treat GBM cells.

### Subcutaneous and Orthotopic Xenograft Studies

The Institutional Animal Care and Use Committee of Southern Medical University approved and supervised all animal procedures. Four‐week‐old female BALB/c nude mice were housed in a pathogen‐free environment under a 12‐h light/12‐h dark cycle, 50% to 70% relative humidity, and a constant temperature of 22–26 °C.

For subcutaneous xenograft experiments, TBD0220TR cells (1 × 10^7^) were subcutaneously injected into the right thigh root of mice. Once the tumor volume reached 50 mm^3^, six mice were randomly assigned to the TMZ group and 6 to the DMSO group. TMZ was administered 5 days a week, at a dose of 66 mg kg^−1^, for three cycles. Following three cycles, the tumor tissue under the skin was removed, chopped into tiny tissue blocks (1 mm^3^), and subcutaneously transplanted into the right thigh root of mice. The experiment was replicated three times.

The 4‐week‐old female BALB/C nude mice were utilized to establish a GBM orthotopic model. Intracranial injection of luciferase lentivirus‐infected TBD0220 cells (1 × 10^5^ cells in 3 µL PBS) was performed under a stereotactic device. Following seven days of injections, TMZ (5 mg kg^−1^) and stiripentol (150 mg kg^−1^) were orally gavaged every day. On days 7, 14, and 21, the tumor development was monitored using an in vivo imaging system (IVIS; PerkinElmer Inc., USA). The survival analysis was performed using Kaplan–Meier curves. The brain tissues were carefully removed four weeks after modeling, preserved in formalin, embedded in paraffin, and subjected to immunohistochemistry (IHC).

### CUT&Tag

For CUT&Tag, the Hyperactive In Situ ChIP Library Prep Kit for Illumina (pG‐Tn5) was utilized in compliance with the manufacturer's guidelines. Briefly, TBD0220 and TBD0220TR cells were bound using concanavalin A‐coated beads. After being resuspended in an antibody buffer, the cells underwent sequential treatment with primary and secondary antibodies directed against H3K9la. The samples and pA‐Tn5 transposase were incubated simultaneously. DNA was isolated, amplified, and purified after transposon activation and tagmentation in order to construct the library. Using VAHTS DNA Clean Beads, purification processes were completed following the construction of the sequencing library. The VAHTS Library Quantification Kit for Illumina was used to quantify the library before being sequenced on an Illumina Nova seq 150PE.

### SLAM‐seq

Using unlabeled culture media, TBD0220 and TBD0220TR cells were grown to the proper cell density. The final concentration of 4‐thiouridine (s4U) in the cell culture media was ensured to reach 100 µM. After 45 min of incubation, the culture medium was immediately removed, the cells were washed twice in PBS, and TRIzol was added to lyse the cells and extract the RNA. The isolated RNA was alkylated using the SLAM‐seq Kinetics kit according to the manufacturer's instructions. Libraries were created from 70 to 200 ng of input RNA using the Quant Seq 3′ mRNA‐Seq Library Prep Kit (REV) for Illumina or the TruSeq stranded mRNA library preparation kit.

### RNA‐seq Analysis

TRIzol was utilized in compliance with the manufacturer's instructions to extract total RNA from TBD0220 and TBD0220TR cells. After that, RNA was digested for reverse transcription, and rRNA was degraded. The Illumina HiSeq4000 platform was used to create and sequence the cDNA libraries. The results of sequencing were mapped to the human reference genome (hg19) using the Hisat2 tool; the DESeq2 R software was used for analysis. The Kyoto Encyclopedia of Genes and Genomes (KEGG) was used for pathway analysis. Biological process (BP) pathway analysis was conducted using Gene Ontology (GO).

### CRISPR/Cas9, Lentivirus, and Transfection

The CRISPR/Cas9 system (GeneChem, China) was employed to produce the stable cell lines LV‐Cas9sgRNA‐LUC7L2. Cas9 and sgRNA lentiviral vectors (LVs) were used to transfect TBD0220TR and U87TR cells. In accordance with the manufacturer's instructions, the stable cell lines were isolated by transfecting the Cas9‐containing LVs into the cells using polybrene. To create the LV‐Cas9‐sgRNA‐LUC7L2 stable cell lines, the LV‐sgRNA‐LUC7L2 were transfected into the TBD0220TR and U87TR cells. Further tests were carried out 48 h post‐LV transfection.

To produce stable overexpression cells, LUC7L2 LVs were transduced into TBD0220 and U87 cells in accordance with the manufacturer's instructions (GeneChem, China). GeneChem produced LUC7L2 N‐terminal truncation mutants (LUC7L2 1–238 AA) and C‐terminal truncation mutants (LUC7L2 239–392 AA). GeneChem produced short hairpin RNAs (shRNAs) that target LDHA, LDHB, and MLH1. Puromycin at a dose of 2.5 mg mL^−1^ was used for two weeks to select transfected cells that tested positive. Quantitative real‐time polymerase chain reaction (qPCR) and Western blot were used to confirm the transfection effectiveness. A summary of the shRNA and sgRNA sequences is shown in Table S4 (Supporting Information).

### Chromatin Immunoprecipitation (ChIP)

Formaldehyde was used to cross‐link TBD0220TR and U87TR cells for 10 min at a final concentration of 1%. The cross‐links were then quenched using a glycine solution. Nuclear lysates were subjected to sonication to digest chromatin. Sufficient lysates, each yielding 100 µg of total protein, were treated at 4 °C overnight with 5 µL of H3K9la antibody and ChIP‐grade magnetic beads. After breaking the cross‐link, the immunoprecipitated and purified samples of DNA were collected using a QIAquick PCR Purification kit. Specific primers were then used for qPCR to measure the samples. SYBR Green Mix was used in qPCR reactions carried out in a thermocycler device. GENEWIZ produced all of the primers. Table S4 (Supporting Information) provides a summary of the ChIP primer sequences.

### RNA Immunoprecipitation (RIP)

The Magna RIPTM RNA‐Binding Protein Immunoprecipitation Kit manufacturer's instructions were followed to conduct the RIP experiment. After extraction, LUC7L2 antibody‐coated magnetic beads were used to incubate cells for a whole night. After numerous rounds of washing with lysis buffer, proteinase K was added to digest the proteins. Following RNA extraction from both the immunoprecipitation and the input, qPCR was used to quantify the amount of MLH1 mRNA normalized to that in the input.

### Cross‐Linked RNA Immunoprecipitation (CLIP)

In order to harvest the relevant cells, UV (254 nm, 400 mJ cm^−2^) was applied. To isolate the nuclei, the final cell pellet was immersed in 4 mL of cell lysis buffer and allowed to sit on ice for 15 min. Subsequently, nuclei were sonicated to yield DNA fragments of 200–1000 base pairs. Following sonication, the chromatins underwent a 30‐min, 37 °C digestion process using 250 U mL^−1^ of DNase; 20 mM of EDTA was used to stop the digestion. Protein‐G‐coated magnetic beads (75 µL) were then mixed with 5 µg of antibody per sample, and the mixture was incubated overnight at 4 °C. After removing the clarified supernatant, 925 µL was added to the antibody‐conjugated beads. The samples were mixed and incubated overnight at 4 °C. The beads were resuspended in 100 µL of NT2 solution supplemented with 30 µg of proteinase K and 1% SDS after being cleaned six times with 1 mL of immunoprecipitation buffer. After that, the samples were shaken for 2 h at 65 °C. Following the manufacturer's recommendations, RNA was extracted using the RNAiso Plus reagent, and random primers were used for reverse transcription. qPCR was used to quantify the specific location of the precipitated MLH1 pre‐mRNA.

### Seahorse XF Glycolysis Stress Assay

The Seahorse XF Glycolysis Stress Test Kit (Agilent, CA) and XF24 Extracellular Flux Analyzer were utilized to determine the extracellular acidification rate (ECAR). In summary, 5 × 10^4^ cells per well were seeded into Seahorse XF24 V7 PS Cell Culture Microplates and cultured overnight. After sequentially injecting glucose (10 mm), oligomycin (1 µm), and 2‐DG (50 mm), ECAR was assessed in an XF base medium supplemented with 2 mm glutamine (pH 7.4).

### RNA Extraction and Quantitative Real‐Time PCR (qPCR)

After total RNA was extracted from cultured cells using TRIzol reagent (Invitrogen, USA), PrimeScript RT Master Mix (Takara Bio, RR036A, Japan) was used to synthesize cDNA in accordance with the manufacturer's instructions. Using an ABI Step One Plus real‐time apparatus (Thermo Fisher Scientific, USA), qPCR was conducted using TB GREEN Premix Ex Taq (Takara Bio, RR420A, Japan), and the results were standardized to the β‐actin level. For quantitative analysis, at least three separate experiments were conducted. Table S4 (Supporting Information) lists the primer sequences.

### Western Blot (WB) Analysis

Using an ice‐cold Whole Cell Lysis Assay (KeyGEN BioTECH, KGP2100, China), proteins were isolated from the cells and then quantified using a BCA assay kit (Beyotime, P0012, China). Protein lysates were separated by SDS‐polyacrylamide gel electrophoresis and subsequently deposited onto polyvinylidene fluoride (PVDF) membranes (Millipore, IPVH00010, USA). After overnight incubation with primary antibodies, the membranes were treated for 1 h with secondary antibodies (Cell Signaling Technology, USA) coupled with horseradish peroxidase (HRP). Using the ECL detection system (Millipore, WBKLS0500, USA), immunoreacted bands were imaged using the ImageQuant LAS500 chemiluminescence (General Electric, USA). Ultimately, ImageJ (National Institute of Health, USA) was utilized to assess protein expression, with β‐actin serving as a loading control. Table S5 (Supporting Information) contains a list of the antibodies used.

### Cell Viability Assay

Cells were seeded in 96‐well plates at a concentration of 5 × 10^3^ cells per well and cultured for 24 h. Next, the medium was replaced with an altered formula. After incubation at 37 °C for 2 h, the viability of treated cells was assessed using the CCK‐8 test (Dojindo, CK04, Japan) in accordance with the manufacturer's instructions. An optical density (OD) reader (BioTek, VT) was used to measure the absorbance at 450 nm.

### Colony Formation Assay

Assays for cell growth and colony formation were conducted. Briefly, 500 cells were plated onto 6‐well plates and cultured for 14 days for the colony formation test. Following a 10‐min room temperature prefixation with 4% paraformaldehyde, the cells were stained for 30 min at room temperature using a crystal violet staining solution (Beyotime, #C0121, China). The samples were imaged under a bright‐field microscope (Olympus Corporation, #CX41, Japan) after being washed with distilled water.

### Flow Cytometry Analysis

Prior to apoptotic evaluation, cells were treated with various compounds in accordance with the instructions provided by the fluorescein isothiocyanate (FITC) Annexin‐V/prodium iodide (PI) apoptosis detection kit. Flow cytometry (BD Biosciences, USA) was used to determine the proportion of apoptotic cells. Detections were performed after 48 h of TMZ or stiripentol treatment on cells.

### Immunofluorescence and Confocal Imaging

Cells that had been grown and preserved on confocal plates were blocked with 5% bovine serum albumin (BSA), permeabilized for 15 min with 0.5% Triton X‐100 (Sigma‐Aldrich, X100, USA), and fixed with 4% paraformaldehyde. After overnight incubation at 4 °C with primary antibodies, an additional 1‐h incubation at room temperature was conducted with secondary antibodies. Nuclear counterstaining was carried out with DAPI (Cell Signaling Technology, #4083, USA). Phaloidin was used to stain intracellular actin (Cell Signaling Technology, #13 054, USA). The cells were imaged with a laser confocal microscope (Nikon, Japan). Assays were performed after 48 h of TMZ or stiripentol treatment on cells.

### Hematoxylin & Eosin (H&E) and Immunohistochemical (IHC) Staining

Paraffin‐fixed tissue samples were sectioned into 4–5 mm pieces, dewaxed, and hydrated. Antigen retrieval was conducted in citrate at 95 °C for 30 min. Sections were subjected to an overnight incubation at 4 °C with primary antibody against pan Kla, H3K9la, LUC7L2, MLH1, Ki67, and γ‐H2AX, followed by a 1‐h room temperature incubation with HRP‐conjugated secondary antibody. Sections were stained with H&E and visualized using diamethoxybenzidine (DAB). A scanner (3DHISTECH, Hungary) was used to obtain digital images, and Image J was used for quantitative analysis.

### Ethics Statement

The medical ethics committee at Zhujiang Hospital granted approval for the acquisition and analysis of all clinical GBM samples (Approval No. 2021‐KY‐129‐01). The informed consent form has been signed by every participant. The Animal Ethical and Welfare Committee granted approval for all animal experiments (Approval No. LAEC‐2023‐070).

### Statistical Analysis

The statistical analysis was conducted utilizing GraphPad Prism 8. Independent‐sample Student's *t*‐test was applied for comparison between two groups. To compare three or more experimental groups, one‐way and two‐way analyses of variance (ANOVA) were performed. In the figures, error bars reflect the mean ± standard deviation (SD). *p* < 0.05 was considered statistically significant: ^∗^ *p* < 0.05, ^∗∗^ *p* < 0.01, ^∗∗∗^ *p* < 0.001, and ^∗∗∗∗^ *p* < 0.0001; ns: not significant.

## Conflict of Interest

The authors declare no conflict of interest.

## Author Contributions

H.G., B.L., and Q.Y. conceptualized and designed the study. Q.Y., Z.W., Y.S., Y.L., X.Z., X.L., T.Y., M.Z., B.Z., T.Z., J.L., and Y.W. conducted the experiments. Q.Y. analyzed the data, produced the figures, and composed the manuscript. All authors approved the final version of the manuscript.

## Supporting information

Supporting Information

## Data Availability

The data that support the findings of this study are available from the corresponding author upon reasonable request.
