# Supplementary material for: Histone H3K9 Lactylation Confers Temozolomide Resistance in Glioblastoma via LUC7L2‐Mediated MLH1 Intron Retention
Source: Adv Sci (Weinh). 2024 Mar 13;11(19):2309290. doi: 10.1002/advs.202309290 (PMC11109612; doi:10.1002/advs.202309290)
Supplement: Supplementary file 1 — Supporting Information [file ADVS-11-2309290-s001.pdf]

ADVANCED SCIENCE
Open Access

Supporting Information

for *Adv. Sci.*, DOI 10.1002/advs.202309290

Histone H3K9 Lactylation Confers Temozolomide Resistance in Glioblastoma via LUC7L2-Mediated MLH1 Intron Retention

Qu Yue, Zhao Wang, Yixiong Shen, Yufei Lan, Xiangyang Zhong, Xin Luo, Tao Yang, Manqing Zhang, Boming Zuo, Tianci Zeng, Jiankun Lu, Yuankai Wang, Boyang Liu and Hongbo Guo**

Supporting Information

Histone H3K9 Lactylation Confers Temozolomide Resistance in Glioblastoma via LUC7L2-mediated MLH1 Intron Retention

Qu Yue, Zhao Wang, Yixiong Shen, Yufei Lan, Xiangyang Zhong, Xin Luo, Tao Yang, Manqing Zhang, Boming Zuo, Tianci Zeng, Jiankun Lu, Yuankai Wang, Boyang Liu, Hongbo Guo**

Q. Yue, Z. Wang, Y. Shen, Y. Lan, X. Zhong, X. Luo, T. Yang, M. Zhang, B. Zuo, T. Zeng, J. Lu, Y. Wang, B. Liu, H. Guo

Department of Neurosurgery Center, The National Key Clinical Specialty, The Engineering Technology Research Center of Education Ministry of China on Diagnosis and Treatment of Cerebrovascular Disease, Guangdong Provincial Key Laboratory on Brain Function Repair and Regeneration, The Neurosurgery Institute of Guangdong Province, Zhujiang Hospital, Southern Medical University, Guangzhou 510282, China

E-mail: lby19881118@163.com, guohongbo911@126.com

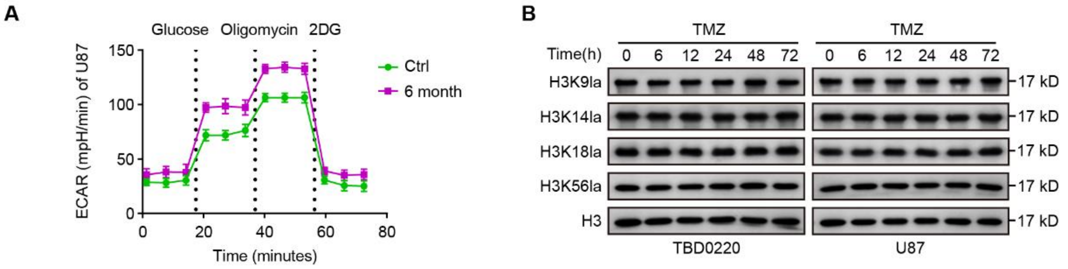

Figure S1

H3K9la is highly expressed in recurrent GBM, and chronic TMZ exposure increases H3K9la. A) ECAR was measured in U87 at indicated time. B) H3K9la, H3K14la, H3K18la and H3K56la was measured by WB in TBD0220 and U87 at indicated time.

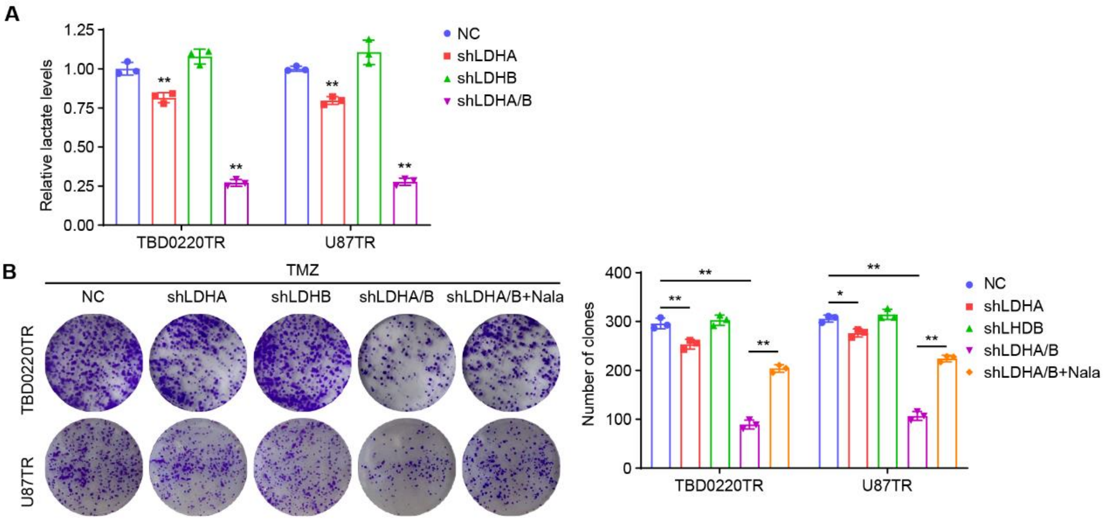

Figure S2

Inhibition of H3K9la enhances GBM TMZ sensitivity. A) Lactate levels were measured after LDHA and LDHB knockdown. B) Colony formation assays were performed after LDHA and LDHB knockdown and Nala addition. All values are presented as the mean ± SD. $^{*}P < 0.05$, $^{**}P < 0.01$, $^{***}P < 0.001$.

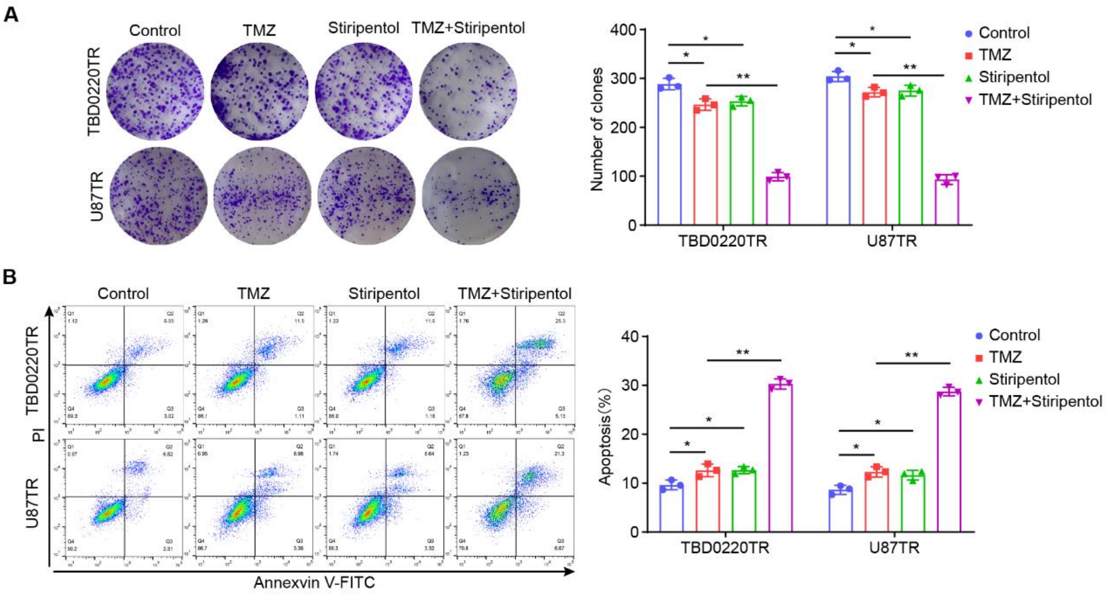

Figure S3

Stiripentol enhances the efficacy of TMZ by inhibiting H3K9la. A) Colony formation assays were performed after stiripentol (500μM) and TMZ (200μM) treatment. B)

Apoptosis rate was measured after stiripentol (500μM) and TMZ (200μM). All values are shown as the mean ± SD. $^{*}P < 0.05$, $^{**}P < 0.01$.

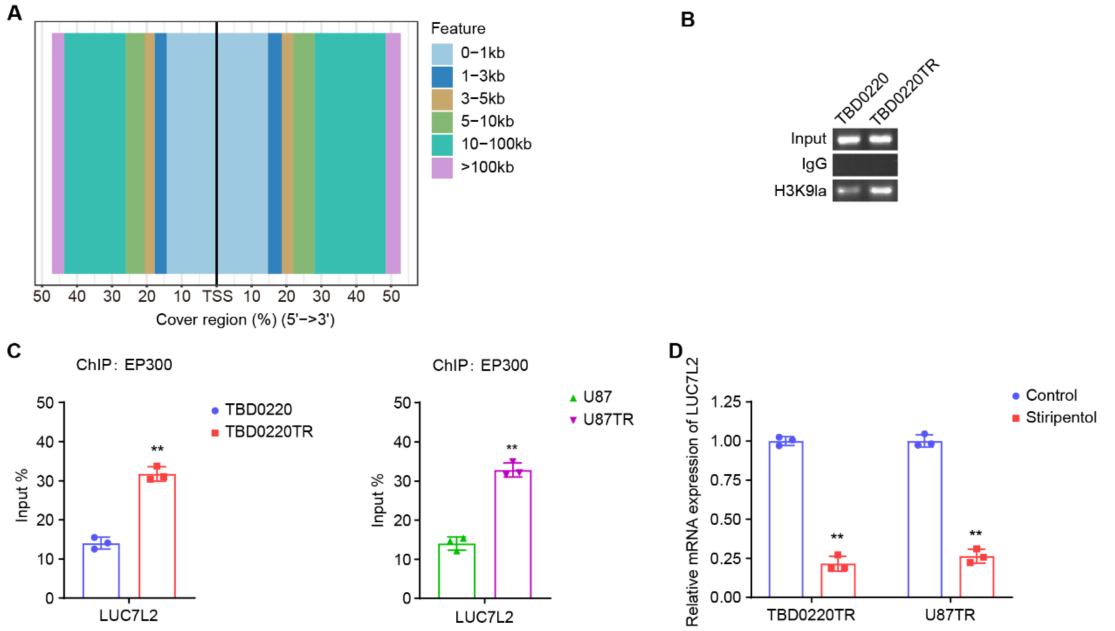

Figure S4

H3K9la activates LUC7L2 transcription. A) Upregulated H3K9la enrichment peaks in TBD0220TR cells. B) Using antibodies against H3K9la, ChIP analysis of the identified promoters was conducted in parent cells and TMZ-resistant cells. C) Using antibodies against EP300, qChIP analysis of the identified promoters was conducted out in parent cells and TMZ-resistant cells. D) LUC7L2 levels were measured by qPCR after trstiripentol (500μM). All values are shown as the mean ± SD. $^{**}P < 0.01$.

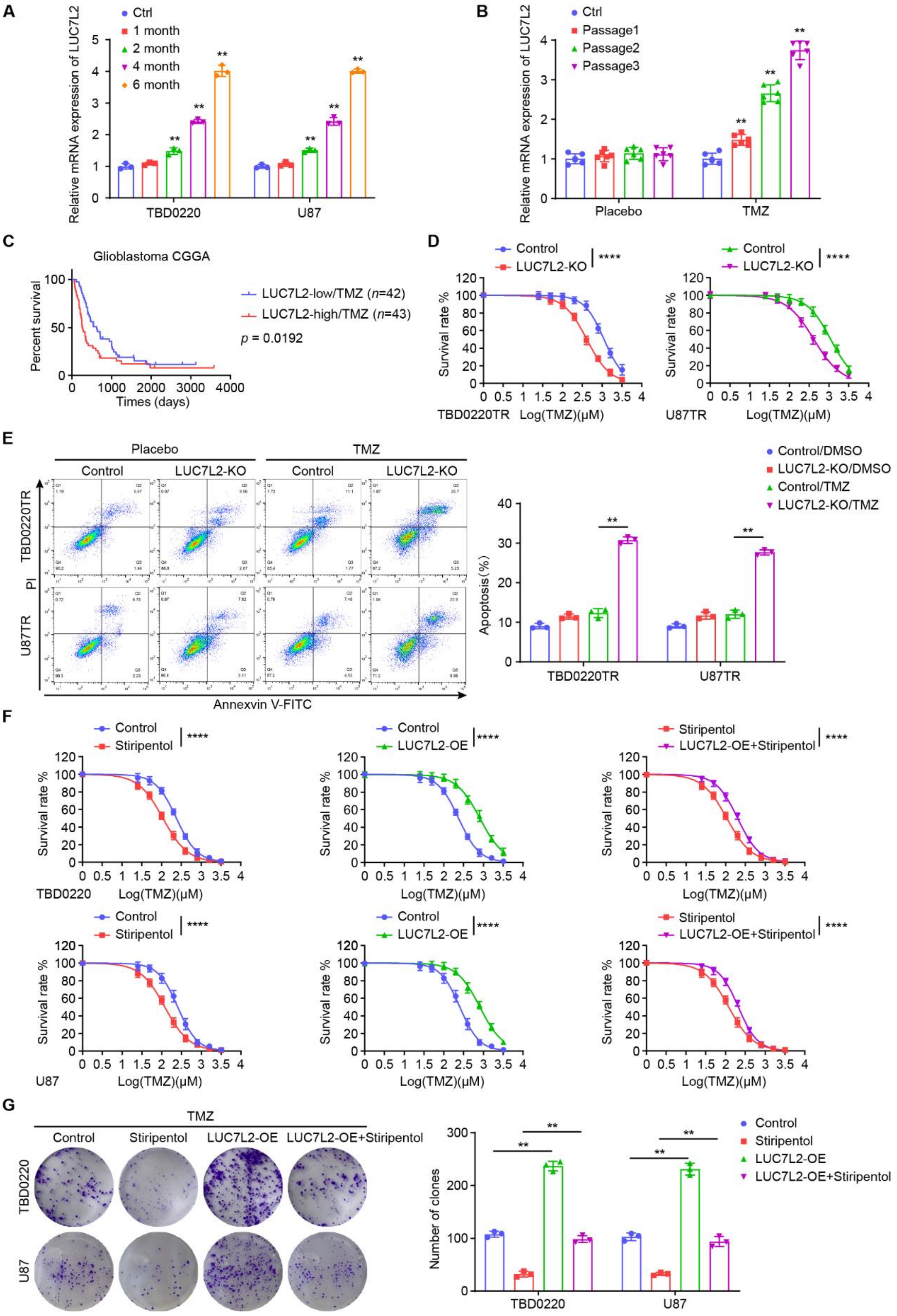
A
B
C
D
E
F
G
Relative mRNA expression of LUC7L2
Ctrl
1 month
2 month
4 month
6 month
TBD0220
U87
Passage1
Passage2
Passage3
Placebo
TMZ
Glioblastoma CGGA
Percent survival
Times (days)
LUC7L2-low/TMZ (n=42)
LUC7L2-high/TMZ (n=43)
p = 0.0192
Control
LUC7L2-KO
Survival rate %
TBD0220TR
U87TR
Log(TMZ)(μM)
Control/DMSO
LUC7L2-KO/DMSO
Control/TMZ
LUC7L2-KO/TMZ
PI
Annexvin V-FITC
Apoptosis(%)
Stiripentol
LUC7L2-OE
LUC7L2-OE+Stiripentol
Number of clones

Figure S5

LUC7L2 promotes TMZ resistance in GBM, and overexpression of LUC7L2 reverses the effect of stiripentol. A) LUC7L2 levels was measured by qPCR in chronic TMZ exposure model of TBD0220 and U87 cells at indicated time. B) qPCR analysis of LUC7L2 of different groups and passages in chronic TMZ exposure model of mice. C) Using data from the GBM CGGA database, Kaplan-Meier graphs highlight the overall survival of patients with high or low expression of LUC7L2 in GBM patients undergoing TMZ treatment. D) Cell survival rate was measured after LUC7L2 knockout. E) Apoptosis rate was measured by flow cytometry after LUC7L2 knockout. F) Cell survival rate was measured with or without LUC7L2 overexpression treated with stiripentol (500μM). G) Colony formation assays were performed with or without LUC7L2 overexpression treated with stiripentol (500μM). All values are shown as the mean ± SD. $^{**}P < 0.01$, $^{****}P < 0.0001$.

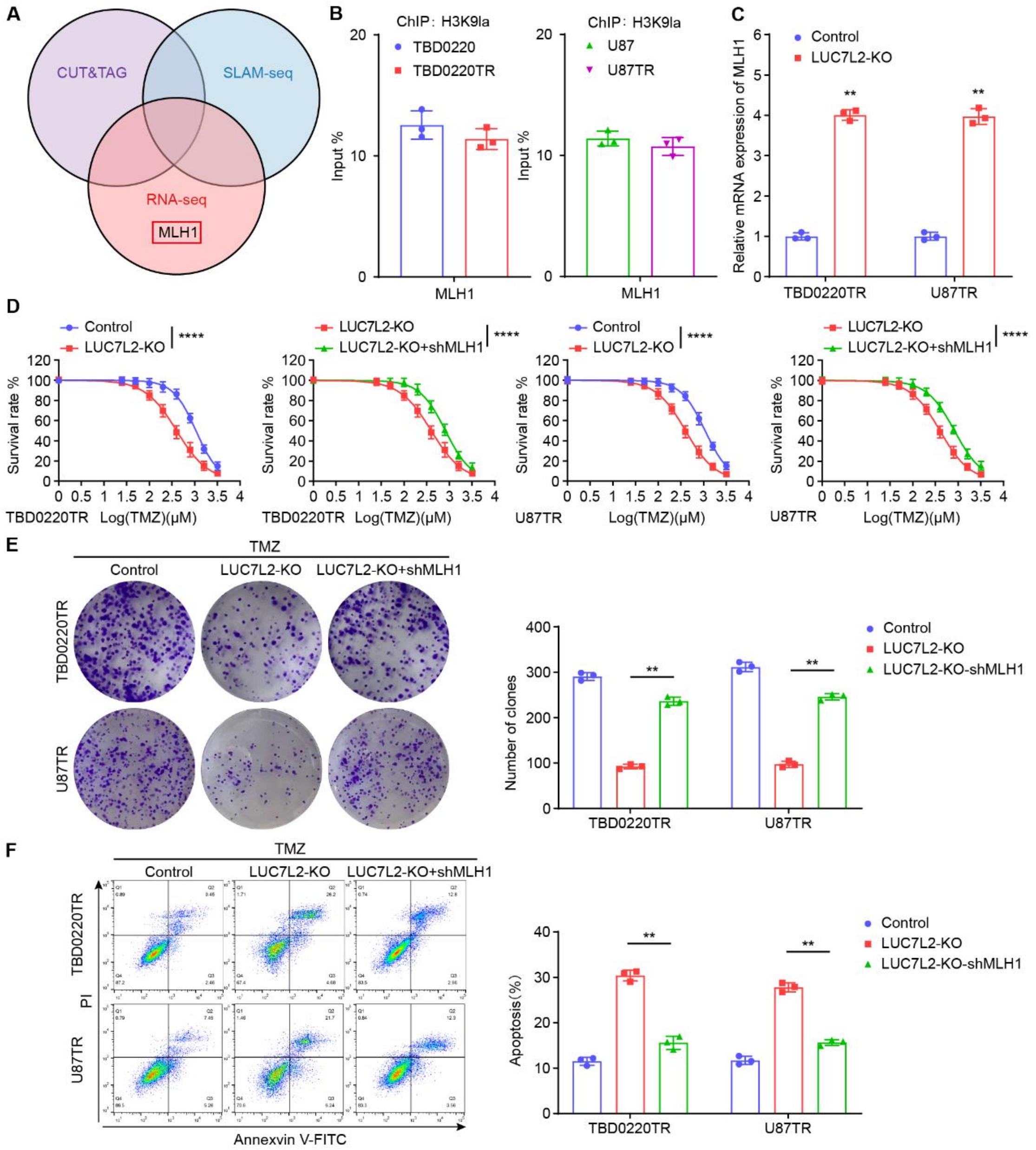

Figure S6

LUC7L2 promotes TMZ resistance by inhibiting MLH1 expression. A) The relationship between MLH1 and RNA-seq, CUT&Tag and SLAM-seq. B) Using antibodies against H3K9la, qChIP analysis of the MLH1 promoters was conducted out in parent cells and TMZ-resistant cells. C) mRNA levels were measured by qPCR with LUC7L2 knockout. D) Cell survival rate was measured with LUC7L2 knockout and MLH1 knockdown. E) Colony formation assays were performed with LUC7L2 knockout and MLH1 knockdown. F) Apoptosis rate was measured with LUC7L2 knockout and MLH1 knockdown. All values are shown as the mean ± SD. $^{**}P < 0.01$, $^{****}P < 0.0001$.

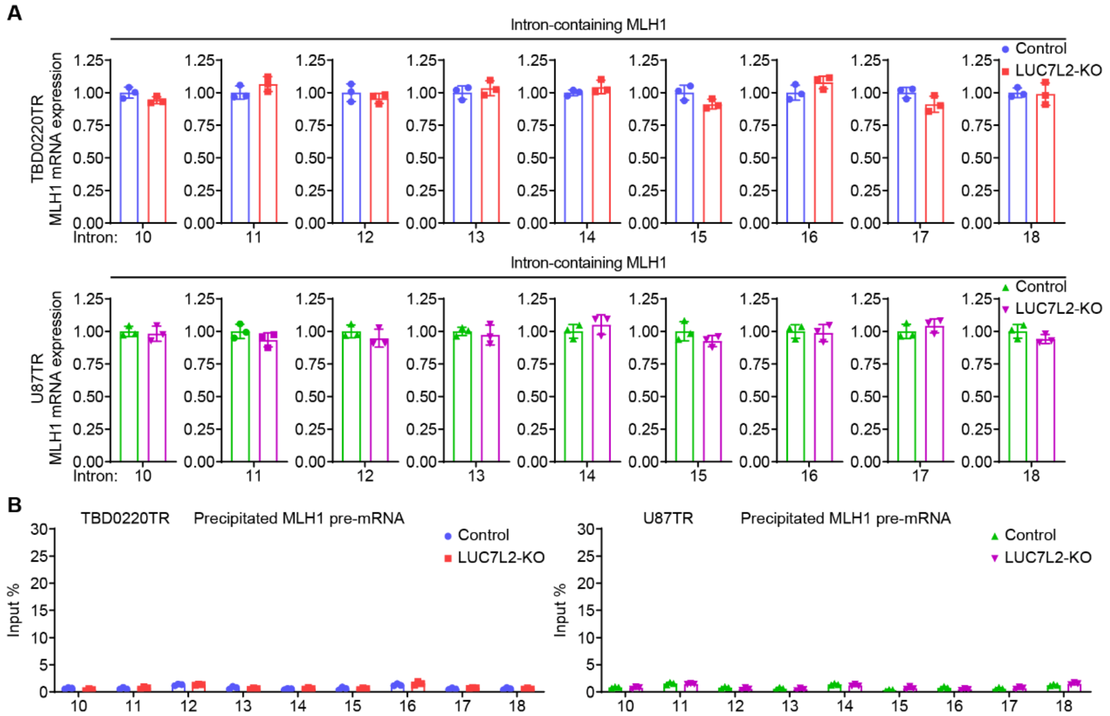

Figure S7

LUC7L2 regulates MLH1 intron 7 retention. A) Influence of LUC7L2- KO on MLH1 transcript levels with retained introns. B) CLIP assays were performed with LUC7L2 antibody, cells were treated with UV (254 nm, 400 mJ cm^{-2}).

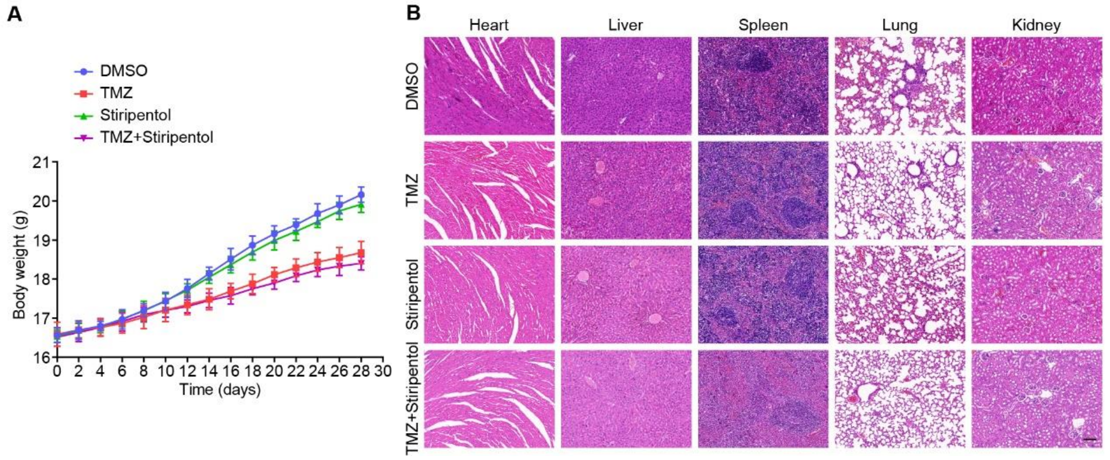

Figure S8

Stiripentol increases TMZ sensitivity in vivo. A) Nude mice were divided into four groups and administered DMSO, TMZ (5 mg kg^{-1}), stiripentol (150 mg kg^{-1}) or TMZ (5 mg kg^{-1}) + stiripentol (150 mg kg^{-1}) by gavage for four weeks. 5 days of TMZ administration followed by two days off, and stiripentol was administered daily. For

four weeks, the body weights of the mice were measured every two days. B) HE staining of major target organs dissected from mice at the end of experiment (28th day). Scale bar, 100 μm.

Table S1. The IC50 (μM) values of TMZ treatment in GBM cells after different treatments.

Group	NC	shLDHA	shLDHB	shLDHA/B	shLDHA/B+Nala
TBD0220TR	1084	806.5	1103	425.7	854.7
U87TR	1066	795.6	1148	406	813.9

Group	Control	LUC7L2-KO
TBD0220TR	1118	410.6
U87TR	1107	416.4

Group	Control	Stiripentol	LUC7L2-OE	LUC7L2-OE +Stiripentol
TBD0220	237.7	108.4	819.2	202.6
U87	254.8	120.2	817.5	210.9

Group	Control	LUC7L2-KO	LUC7L2-KO+shMLH1
TBD0220TR	1072	400.9	825.7
U87TR	1085	401.4	839.2

Table S2. Blood routine analysis in blood samples of mice after 4 weeks continuous treatment.

**Group**	**WBC (10^9 L^{-1})**	**Gran% (%)**	**RBC (10^{12} L^{-1})**	**HGB (g L^{-1})**	**PLT (10^9 L^{-1})**
**DMSO**	**6.81±0.74**	**19.50±0.30**	**9.47±0.34**	**147.05±7.81**	**1078.95±29.41**
**TMZ**	**9.52±0.92**	**15.32±0.85**	**9.54±0.30**	**141.26±1.86**	**1220.91±37.92**
**Stiripentol**	**8.40±0.78**	**18.97±1.56**	**9.70±0.47**	**142.26±3.16**	**1144.16±63.36**

TMZ+Stiripentol	9.80±0.55	15.77±0.84	9.35±0.36	139.37±7.78	1094.95±56.75

Table S3. Values of serum enzymes in blood samples of mice after 4 weeks continuous treatment.

Group	ALT (U L^{-1})	AST (U L^{-1})	T-Bil (μmol L^{-1})	D-Bil (μmol L^{-1})	ALB (g L^{-1})	UREA (mmol L^{-1})	CREA (μmol L^{-1})
DMSO	58.76±1.38	169.38±4.70	1.94±0.23	0.98±0.19	29.89±1.96	11.84±1.47	27.72±1.90
TMZ	53.91±2.51	174.31±6.55	2.29±0.93	0.87±0.17	26.91±2.05	10.64±1.88	28.62±1.94
Stiripentol	56.69±1.62	161.95±5.62	2.61±0.99	0.95±0.12	27.87±1.62	11.39±1.75	27.71±1.52
TMZ+Stiripentol	53.72±2.96	158.96±7.10	2.13±0.28	0.82±0.21	26.37±1.57	9.57±1.30	29.51±1.91

Table S4. Primer sequences and shRNAs used in this study.

Primer name		Sequence (5'-3')
LUC7L2	Forward	GAGAAAGCACGGGCAAAGAAAAGAG
	Reverse	AATAGGCAGAGCAGACTTCACAGAC
PDE4DIP	Forward	AGGTGTTACGCAGTCGGCTAG
	Reverse	GTGCTTGTATCTTCGGTGTCATCC
PMEPA1	Forward	TGCCTGCTGAGCCACTACAAG
	Reverse	GATTCCGTTGCCTGACACTGTG
WIPF1	Forward	CCCTCCTCCTCCTCAGAACAAC
	Reverse	GTCATTGCCGCTGGAACTTGG
KCTD12	Forward	GTGGCTCCTCGTCGTCCTC
	Reverse	GGCTGCTGCTGCGTGAAC
PAPOLA	Forward	AACACAACCGCCACAGAAGC
	Reverse	GTAAGTACGCAGTCAGTCTCCTTG
CNIH1	Forward	GGCTTACACTGGGTCTCAATATGC
	Reverse	ACCATCCTTCCTTCTGACAATATGC
PDZRN3	Forward	GCTGGATGAGGGCTGGATGG
	Reverse	CGTCTTCGTCGTGCTTCTTCTG

CBFB	Forward	GCAGGGAGAACAGCGACAAAC
	Reverse	CCAGCCTTTCCAGATAACACAGAC
TOB1	Forward	AACCTCGGCTTGAATGTGAATGAC
	Reverse	TGTGGCTGCTGCTGGCTAC
ASPH	Forward	CCAAGAGCAGCGGCAACAG
	Reverse	TGAAGAATGAAGTTCCTGAGAGTCC
ARHGEF3	Forward	CAGATCAGCAGCACTTGGAAGAAG
	Reverse	GCGGCATTCAGATTCACCAGTC
UQCC1	Forward	GCGTTGCTGGTGCGAGTC
	Reverse	TGGGTAGGAGACACAGGTATCAATC
FRMD4A	Forward	GCACTATCACCGCAACGACTATG
	Reverse	CGCTTGTGGCTGCTGGAATG
MLH1	Forward	TCTCAGGTTATCGGAGCCAGCAC
	Reverse	ATCTTCCTCTGTCCAGCCACTCTC
MLH1 pre-mRNA	Forward	GCTCCATTTGGGGACCTGTAT
	Reverse	CCAGCAAGTCCTGGTAGCAA
MLH1 intron 1	Forward	CGGCCAGCTAATGCTATCAA
	Reverse	GGCAAAGAGGCGTGTCC
MLH1 intron 2	Forward	TGAAGTTGATTCAGATCCAAGACA
	Reverse	AAAGGTCCTGACTCTTCCATGA
MLH1 intron 3	Forward	TTGAGGATTTAGCCAGTATTTCTACC
	Reverse	TGACAGACAATGTCATCACAGG
MLH1 intron 4	Forward	AACAGCTGATGGAAAGTGTGC
	Reverse	AACACTGGTGTTGAGACAGGATTA
MLH1 intron 5	Forward	CAATCAAGGGACCCAGATCA
	Reverse	TTACCCTGAAAACTTAGAAGCAATTT
MLH1 intron 6	Forward	TCCAAGTGAAGAATATGGGAAAA
	Reverse	TGAGCACTAGAACACATTACTTTGATG
MLH1 intron 7	Forward	AGTACACAATGCAGGCATTAGTTTCT

	Reverse	CCTTATCTCCACCAGCAAACTATTAAA
MLH1 intron 8	Forward	TCCATCTTTGGAAATGCTGTT
	Reverse	TTCCAAAATAATGTGATGGAATGA
MLH1 intron 9	Forward	CTACTCAGTGAAGAAGTGCATCTTCTTACT
	Reverse	ATGGATCAGAAATTTTTCCATGGT
MLH1 intron 10	Forward	CCCAAAAACACACACCCATT
	Reverse	CTTTCAAAGAGGAGAGCCTGAT
MLH1 intron 11	Forward	CTCCTGGGCTCCAATTCCT
	Reverse	CAAAAATCTGGGCTCTCACG
MLH1 intron 12	Forward	GAGGACCTACTTCCAGCAACC
	Reverse	TCATGAAAAGCCAAAGTTAGAAGG
MLH1 intron 13	Forward	CTCCAGGAAGAAATTAATGAGCA
	Reverse	CATGACTGCTTTCTCCATTTCC
MLH1 intron 14	Forward	CCTTCTCAACACCACCAAGC
	Reverse	TCCTTAGCTTTTGTGCCTGTG
MLH1 intron 15	Forward	TTATGATTTTGCCAATTTTGGTG
	Reverse	GGAGAGCTACTATTTTCAGAAACGAT
MLH1 intron 16	Forward	CTGAGATGCTTGCAGACTATTTCTC
	Reverse	CACCCGGCTGGAAATTTTAT
MLH1 intron 17	Forward	GGAGGGACTGCCTATCTTCA
	Reverse	TCCAGATCAAAGGGTGGTCA
MLH1 intron 18	Forward	AGGAGTCGACCCTCTCAGG
	Reverse	GATGGGCAAGTTTCATCTCC
LUC7L2 for ChIP	Forward	TACTTTCCGCCCAGGCTAAC
	Reverse	GAGGCGCCAAACCTTACATT
LDHA shRNA		CGAAGACAAATTGAAGGGAGA
LDHB shRNA		CGTGATTGGAAGTGGATGTAA
LUC7L2 sgRNA		CATAATCCGCTCTTAAAGCC
MLH1 shRNA		GCCTGATCTATACAAAGTCTT

Table S5. Primary antibodies and secondary antibodies used in this study.

Antigen	Manufacturer	Catalog Number	Application
Pan Kla	PTM BIO	PTM-1401RM	1:1000 for WB;<br>1:100 for IHC;<br>1:100 for IF;
H3K9la	PTM BIO	PTM-1419RM	1:2000 for WB;<br>1:200 for IHC;<br>1:50 for IF;<br>6μg / 5×10^6 cells for Cut&Tag
H3K14la	PTM BIO	PTM-1414RM	1:2000 for WB
H3K18la	PTM BIO	PTM-1406RM	1:2000 for WB
H3K56la	PTM BIO	PTM-1421RM	1:2000 for WB
H3	Cell Signaling Technology	14269	1:1000 for WB
β-actin	Cell Signaling Technology	4970	1:1000 for WB
LDHA	Cell Signaling Technology	3582	1:1000 for WB
LDHB	Proteintech	66425-1-Ig	1:20000 for WB
γ-H2AX	Cell Signaling Technology	9718	1:100 for IF
LUC7L2	ABclonal	A13096	1:1000 for WB;<br>1:100 for IF;<br>1:200 for IP
MLH1	Abcam	ab92312	1:2000 for WB;<br>1:100 for IF
Anti-rabbit IgG, HRP-linked Antibody	Cell Signaling Technology	7074	1:3000 for WB;
Anti-mouse IgG, HRP-linked Antibody	Cell Signaling Technology	7076	1:3000 for WB
